# A comprehensive analysis of COVID-19 nonlinear mathematical model by incorporating the environment and social distancing

**DOI:** 10.1038/s41598-024-61730-y

**Published:** 2024-05-28

**Authors:** Muhammad Riaz, Kamal Shah, Thabet Abdeljawad, Inas Amacha, Asma Al-Jaser, Manar Alqudah

**Affiliations:** 1 Department of Mathematics, University of Malakand, Dir(L), 18000 Khyber Pakhtunkhwa Pakistan; 2 Department of Mathematics and Sciences, Prince Sultan University, P.O. Box 66833, 11586 Riyadh, Saudi Arabia; 3 Department of Medical Research, China Medical University, Taichung, 40402 Taiwan; 4 Department of Mathematical Science, College of Science, Princess Nourah bint Abdulrahman University, P.O. Box 84428, 11671 Riyadh, Saudi Arabia

**Keywords:** Infection, Mathematical modeling, Stability analysis, Sensitivity, NSFD method, Biological techniques, Computational biology and bioinformatics, Immunology, Zoology, Biogeochemistry, Mathematics and computing

## Abstract

This research conducts a detailed analysis of a nonlinear mathematical model representing COVID-19, incorporating both environmental factors and social distancing measures. It thoroughly analyzes the model’s equilibrium points, computes the basic reproductive rate, and evaluates the stability of the model at disease-free and endemic equilibrium states, both locally and globally. Additionally, sensitivity analysis is carried out. The study develops a sophisticated stability theory, primarily focusing on the characteristics of the Volterra–Lyapunov (V–L) matrices method. To understand the dynamic behavior of COVID-19, numerical simulations are essential. For this purpose, the study employs a robust numerical technique known as the non-standard finite difference (NSFD) method, introduced by Mickens. Various results are visually presented through graphical representations across different parameter values to illustrate the impact of environmental factors and social distancing measures.

## Introduction

The new coronavirus SARS-CoV-2, which gave rise to COVID-19, first appeared in Wuhan, China, at the end of 2019 and quickly spread throughout the world. It dispersed throughout continents, having a profound effect on social, economic, and health issues globally. When an infected person coughs, sneezes, talks, or breathes, respiratory droplets are the main way that the virus spreads. It can cause minor respiratory problems, acute respiratory distress syndrome (ARDS), severe pneumonia, and in rare cases, even death. Severe illness is more common in some populations, such as the elderly and people with underlying medical issues. We refer for details some work as^1,2^.

Mathematicians have played a pivotal role in studying COVID-19 by employing diverse strategies, including the development of intricate epidemiological models such as susceptible, infected, and recovered (SIR) and susceptible, exposed, infected, and recovered (SEIR) utilizing statistical analysis and machine learning for data interpretation and forecasting, applying optimization techniques to devise control measures and vaccination strategies, understanding network dynamics through network theory to identify transmission patterns, and developing mathematical immunology models to predict vaccine efficacy and immune response dynamics. Collaborating across disciplines, mathematicians have worked closely with epidemiologists, virologists, healthcare professionals, and policymakers to integrate mathematical models with empirical data, guiding public health responses and aiding policymakers in making informed decisions to effectively mitigate the spread of the virus and ultimately save lives. In this context, reference is made to the works cited as^3–6^.

Mathematical modeling stands out as the most effective means of predictive analysis for accurately anticipating the prevalence of infectious diseases. By forecasting infection rates, valuable insights can be gained to formulate effective strategies for disease control. For instance, researchers done some important work referenced as^7–9^. The field of mathematical models for infectious diseases has undergone rapid advancement in recent decades, a progress attributed in part to the enhanced capacity of researchers to gather relevant data^10–12^.

An essential focus in epidemiology revolves around examining the global stability of endemic equilibrium within the mathematical models of infectious diseases. Researchers established numerous results on the global and local stability analysis. We refer some sophisticated results as^13–16^. Researchers have introduced various methods to establish the stability of equilibrium points, including monotone dynamical systems, the geometric approach, and the application of Lyapunov functions. We refer to the significant contributions of researchers as presented in^17–19^. For more informative contribution about stability, readers should read the articles like^20,21^. The V–L approach is a mathematical framework used to analyze the dynamics of complex systems, including infectious disease models like those used in studying COVID-19. We refer some references as^22,23^. The study of infectious diseases like COVID-19 typically involves mathematical modeling to understand how the disease spreads through populations. Differential equation models, such as compartmental models (e.g., SIR, SEIR models), are more commonly used in epidemiology to predict disease dynamics and assess the impact of interventions. For reader, we refer to^24–26^.

However, the V–L approach, originating from fields like control theory and nonlinear dynamics, can potentially offer insights into the stability and behavior of dynamical systems. In the context of infectious diseases like COVID-19, this approach could potentially be used to analyze the stability of disease equilibria, study the effects of parameter variations or interventions on disease dynamics, and understand the resilience or vulnerability of the system to perturbations. Researchers continuously explore new mathematical and computational techniques to enhance our understanding of infectious diseases, and the V–L approach might be an area of interest for more in-depth investigations in the future to gain insights into the dynamics of diseases like COVID-19. Authors^27^ introduced a mathematical model for an infectious disease . They analyzed the stability of both the disease-free and endemic equilibria, considering fixed controls. Additionally, they explored optimal control strategies for the disease, incorporating time-dependent controls. Liao and Wang^28^ proposed a method for ensuring global stability of the endemic equilibria, employing a combination of the Lyapunov function method and V–L matrices properties. However, this approach faced challenges, such as determining suitable Lyapunov functions and coefficients.

In our study, we propose a modification to the Lyapunov function method, integrating the theory of V–L stable matrices^28–30^. The key distinction lies in our modified method’s repeated use of Lemma 2.4 and Lemma 2.8 (presented in^28^), along with the reduction of matrix dimensions. This departure from the original method, which sometimes omitted this technique, shifts the analysis from differentiable functions to matrices associated with them. Notably, our modified approach leverages Lemma 2.4, Lemma 2.8, and matrix dimension reduction in each step, simplifying the implementation of higher-dimensional matrices. This reduction in complexity can enhance the computational efficiency compared to the original method^28^.

Moreover, the authors in^27^ employed intricate concepts and theorems to establish the global stability of the endemic equilibrium. In contrast, Tian and Wang^31^ investigated the global stability of cholera epidemic models using a modified approach. They relied on monotone dynamical systems, the geometric approach, and V–L stable matrices. Their models encompassed various functions, transmission pathways, and pathogen growth rates. The modified method presented here offers a straightforward means of proving the global stability of models featuring nonlinear incidence rates. Numerical analysis is performed by using NSFD method given by Mickens^32^. The said tool is a powerful numerical method which has more advantages than usual Euler and RK4 method. The dynamical properties positivity of the aforesaid method has been proved. We refer fundamental work on the said method as^33,36^. To thoroughly explore the application of fraction calculus in disease modeling trends, we consult the works cited in^37–39^. Moreover, to extend the use of fractional calculus from HIV and dengue dynamics to COVID-19, incorporating environmental factors and social distancing measures for a comprehensive analysis of disease transmission and control interventions. We consult the work cited in^40–43^. By applying fractional calculus to our COVID-19 research work, we gain a deeper understanding of the epidemic’s dynamics, leading to more accurate predictive models and better strategies for control and mitigation. For this, we reference the works cited in^44–47^.

Here, the following question arises:

1. Why is COVID-19 timeliness to explore such a study?
2. What makes this study different from the previously published COVID-19 studies?
3. Are there any similarly findings in line with the previously published studies?

Our analysis answers the first question as this study explores COVID-19 urgently due to its global impact, necessitating a deeper understanding to inform effective intervention strategies. And answers the second question as this study differs from previous COVID-19 research by proposing a novel modification to stability analysis methods, integrating V–L stable matrices, offering a more efficient approach to analyzing disease models.

Researchers from various fields aim to enhance understanding and support medical researchers in responding effectively to both current and future pandemics. In this context, mathematical researchers utilize previous data to modify and advance models, providing deeper insights into pandemics. Therefore, this research gives the answer of the third question as, our study involves the utilization of mathematical models, including SIR and SEIR models, to analyze disease dynamics and assess interventions. It focuses on global stability analysis within infectious disease models and advancements in methodologies. The application of numerical analysis methods, such as the NSFD method, is employed for studying disease dynamics and ensuring numerical stability.

We introduce Table 1 summarizing the primary findings from relevant literature sources that we have incorporated into our research. This table offers a concise summary of the literature sources supplying the essential results cited in this paper. By having this table, readers could quickly glance at it to understand the main findings of each literature source without having to dig through the main text for that information. This approach helps to organize and present the information in a more digestible format, enhancing the overall readability and accessibility of the document.

**Table 1:** Summarization of the key results of each literature.

Year	Key data sources highlighting our research findings
2021	Chien and Shateyi^30^, primarily employed global stability analysis using V–L matrices to investigate the transmission dynamics of Babesiosis
2021	Shao and Shateyi^49^, employed mathematical modeling, specifically utilizing Lyapunov functions and V–L matrices, to analyze the global stability of the SEIRS epidemic model with a non-linear incident rate, and validated the findings through numerical simulations
2020	Mwalili et al.^1^, utilized mathematical modeling, particularly a modified SEIR compartmental model, alongside numerical methods like fourth and fifth order Runge-Kutta techniques, to forecast COVID-19 epidemic patterns and analyzed the basic reproduction number using the next generation matrix approach
2020	Masoumnezhad et al.^50^, studied global stability analysis by utilizing a modification of the V–L matrix method, which combines Lyapunov functions and V–L matrices to analyze the mathematical model of an infectious disease
2018	Verma and Kayenat^32^, analyzed and established convergence of Mickens’ type non-standard finite difference (NSFD) schemes, applied them to Lane-Emden equations, and compared the numerical results with existing analytical solutions and standard finite difference (FD) schemes, showing NSFD’s ability to efficiently approximate solutions near singular points where FD fails
2017	Zahedi and Kargar^48^, used a methodologies involved mathematical modeling of HIV/AIDS transmission dynamics, incorporating constant controls, V–L stable matrices, and numerical simulations to analyze global stability
2012	Mickens and Washington^36^, created an NSFD scheme for an SIRS model of respiratory virus transmission, preserving conservation laws and ensuring positive solutions for all time step sizes
2012	Liao and^28^, employed mathematical epidemiological models integrating human and environmental factors, using V–L stable matrices alongside classical Lyapunov functions to analyze global stability, yielding new results for three- and four-dimensional systems, validated via numerical simulations

**Manuscript Organization:** Section "Introduction" contains literature review. Section "Research and data methodology" contains formulation of the model. Moving to section "Local stability of DFE and EE", we assess the disease free equilibrium (DFE) and endemic equilibrium (EE) points, local stability of the model. Section "Global stability of DFE and EE" provides a brief background essential for establishing the global stability of the model. Section "Sensitivity analysis" we present sensitivity of the model. Section "Numerical results and discussion" contains numerical analysis and discussion. Section "Conclusion" contains conclusion.

## Research and data methodology

In epidemiology, comprehending the dynamics of infectious diseases is paramount for effective public health interventions and policy formulation. Mathematical models serve as indispensable tools in simulating and predicting disease spread within populations. The model described herein, represented by the system of differential equations in Eq. (2), embodies a compartmental model widely utilized in epidemiology known as the SEIR model. This model has been extended to encompass separate compartments for asymptomatic and symptomatic infections, prophylactic treatment, and the pathogen population.

In this section, we outline the investigation conducted by authors referenced in^1^ on the subsequent mathematical framework given by the following system.

1 $$\begin{aligned} S^{\prime }&=b-\frac{\beta _{1}SP}{1+\alpha _{1}P}-\frac{\beta _{2} S(I_{A}+I_{S})}{1+\alpha _{2}(I_{A}+I_{S})}+\varphi E-\mu S,\\ E^{\prime }&=\frac{\beta _{1}SP}{1+\alpha _{1}P}+\frac{\beta _{2} S(I_{A}+I_{S})}{1+\alpha _{2}(I_{A}+I_{S})}-(\varphi +\mu +\upsilon )E,\\ I^{\prime }_{A}&=(1-\eta )\upsilon E-(\mu +\delta +\gamma _{A})I_{A},\\ I^{\prime }_{S}&=\eta \upsilon E-(\mu +\delta +\gamma _{S})I_{S},\\ R^{\prime }&=\gamma _{A}I_{A}+\gamma _{S}I_{S}-\mu R,\\ P^{\prime }&=\tau _{A}I_{A}+\tau _{S}I_{S}-\mu _{P}P. \end{aligned}$$

### Model building process

Upon a comprehensive review of the research paper as cited above, we came to the conclusion that their suggested model was not adequately scrutinized and needs further investigation and analysis. We refined the model by reverting the recovered class into the susceptible class due to considerations regarding the weakened immune system, and subsequently made additional adjustments to the model outlined in system (1). We then revisit the updated model given in (2) and establish a detailed analysis for the stability and numerical results by using NSFD method.

#### Compartmental structure

**Figure 1:**
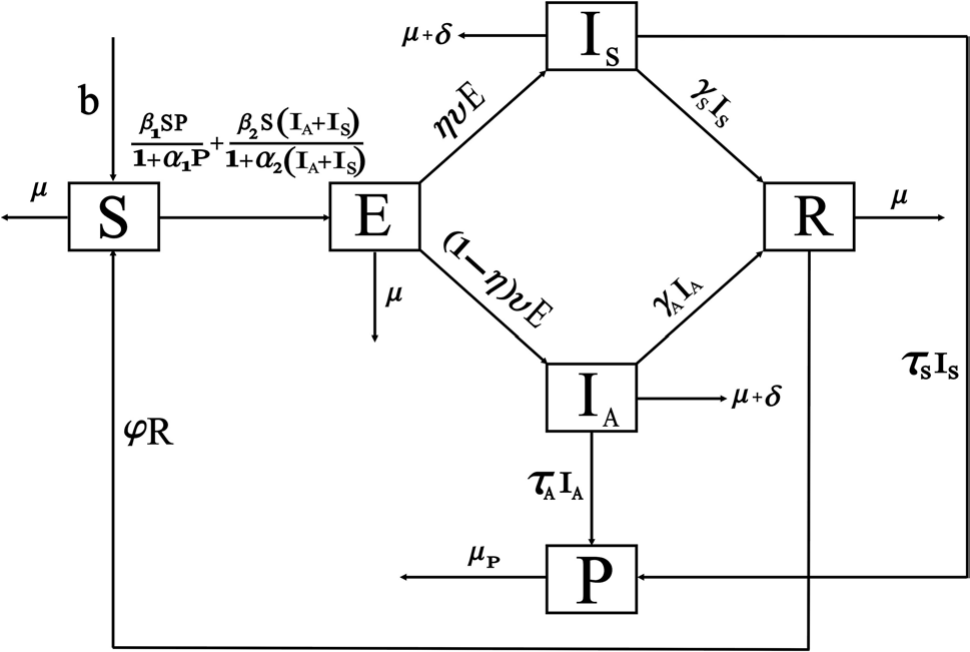
Schematic diagram of our proposed model (2).

The population is compartmentalized based on disease status and the pathogen population into susceptible (*S*), exposed (*E*), symptomatic infectious ($I_{S}$), asymptomatic infectious ($I_{A}$), recovered (*R*), and pathogen (*P*) categories, defined in Table 2. A graphical representation illustrating the proposed model governing the system of differential equations outlined in (2) is shown in Fig. 1.

Each compartment is governed by a differential equation describing the rate of change of individuals or the pathogen population over time. Susceptible individuals can transition to exposed through contact with infected individuals or the pathogen, influenced by transmission dynamics. Exposed individuals progress to infectious states, symptomatic or asymptomatic, at specific rates. Recovery and disease-induced mortality rates determine transitions from infectious to recovered states. Pathogen population dynamics are affected by transmission from infectious individuals and decay processes.

The model integrates parameters governing disease transmission, progression rates, recovery rates, mortality rates, and pathogen dynamics nomenclature is given in Table 2. This model construction process allows for a comprehensive representation of infectious disease dynamics, facilitating insights into transmission patterns, intervention strategies, and disease control measures.

The dynamic of the refined model governs in the form of differential equations given in (2).

2 $$\begin{aligned} S^{\prime }&=b-\frac{\beta _{1}SP}{1+\alpha _{1}P}-\frac{\beta _{2} S(I_{A}+I_{S})}{1+\alpha _{2}(I_{A}+I_{S})}+\varphi R-\mu S,\\ E^{\prime }&=\frac{\beta _{1}SP}{1+\alpha _{1}P}+\frac{\beta _{2} S(I_{A}+I_{S})}{1+\alpha _{2}(I_{A}+I_{S})}-(\mu +\upsilon )E,\\ I^{\prime }_{S}&=\eta \upsilon E-(\mu +\delta +\gamma _{S}+\tau _{S})I_{S},\\ I^{\prime }_{A}&=(1-\eta )\upsilon E-(\mu +\delta +\gamma _{A}+\tau _{A})I_{A},\\ R^{\prime }&=\gamma _{A}I_{A}+\gamma _{S}I_{S}-(\mu +\varphi ) R,\\ P^{\prime }&=\tau _{A}I_{A}+\tau _{S}I_{S}-\mu _{P}P. \end{aligned}$$

In summary, our model offers a robust framework for studying and understanding the complexities of COVID-19 dynamics, providing valuable insights for informing public health policies and interventions.

### Model testing approach

To ensure the reliability and applicability of our proposed model, we conducted a rigorous testing process, employing various methodologies to assess its predictive capabilities and robustness. The testing framework comprised the following:

The model’s predictive accuracy has been validated by comparing its simulations with past epidemiological data obtained from^1^. Through ongoing adjustments to the model’s parameters, we ensured its close alignment with observed trends in disease incidence, prevalence, and other relevant metrics. This process confirmed the reliability of our model in forecasting epidemiological outcomes.

**Table 2:** Physical illustration of nomenclature of the model (2).

Nomenclature	Description
*S*	Uninfected (susceptible) class
*E*	Exposed class
$I_{S}$	Infected symptomatic infected comartment
$I_{A}$	asymptomatic infected compartment
*R*	Recovered compartment
*P*	Compartment of organism that causes disease
*b*	The rate of childbirth in the human population
$\mu$	Natural death of human
$\mu _{P}$	Pathogen natural mortality rate in the environment
$\upsilon$	Migration rate from exposed compartment to $I_{S}$ and $I_{A}$
$\eta$	Saturation constant
$\delta$	Death due to corona
$\alpha _{1}$	Extent of contact with a contaminated environment
$\alpha _{2}$	Degree of contact with an infectious person
$\beta _{1}$	Transmission rate from *S* to *E* through pathogen contact
$\beta _{2}$	Transmission rate from *S* to *E* through contact with $I_{A}$ and/or $I_{S}$ individuals.
$\gamma _{S}$	Migration rate of the recovered individuals from $I_{S}$ to *R*
$\gamma _{A}$	Migration rate of the recovered individuals from $I_{A}$ to *R*
$\tau _{S}$	Migration rate of pathogens from $I_{S}$ to *P*
$\tau _{A}$	Migration rate of pathogens from $I_{A}$ to *P*
$\varphi$	Migration rate due to weak immune system from *R* to *S*

A comprehensive sensitivity analysis has been conducted to evaluate the impact of parameter variations on model outcomes. By systematically varying key parameters within plausible ranges, we assessed the sensitivity of the model to different inputs and identified parameters with the most significant influence on model predictions. This analysis enabled us to better understand the model’s behavior under varying conditions and to identify potential sources of uncertainty.

Finally, to ensure the robustness and credibility of our model. The feedback received from peer reviewers strengthened the validity and reliability of our model and its implications for public health policy and practice.

### Feasibility of solution

After describing the human population in system (2), it is imperative to demonstrate that, for all $t\ge 0$, the state parameters $S, E, I_{A}, I_{S}, R, and P$ are positive. For every $t\ge 0$, the solution with positive beginning data remains positive and is bounded. Now from system (2), clearly one has that $\frac{dN}{dt}=b-\mu N-\left((\delta -\tau _{S})+(\delta -\tau _{A})+\mu _{P}P\right)$ and $\sup _{t\longrightarrow +\infty } N\le \frac{b}{\mu }$. The feasible region is described as

3 $$\begin{aligned} \Omega =\left\{ S, E, I_{A}, I_{S}, R, P\in \mathbb{R}^{6}_{+}:0\le N\le \frac{b}{\mu }\right\} . \end{aligned}$$

At this point, (3) is positively invariant with respect to (2). This indicates that all solutions of the system with $(S, E, I_{A}, I_{S}, R, P)\in R^{6}_{+}$ remain in $\Omega$ and thus the suggested model (2) is epidemiologically well formulated.

DFE

Solving equations given in system (2) we get the DFE points by setting $S^{\prime }$, $E^{\prime }$, $I^{\prime }_{A}$, $I^{\prime }_{S}$, $R^{\prime }$ and $P^{\prime }$ all equal to zero. In this case $I^{\prime }_{A}=I^{\prime }_{S}=P^{\prime }=0$, which implies that $E=0$ and $R=0$ too. Hence, we get the DFE $E_{0}=(\frac{b}{\mu },0,0,0,0,0)$.

EE

To deduce EE point, let use $S^{\prime }$, $E^{\prime }$, $I^{\prime }_{A}$, $I^{\prime }_{S}$, $R^{\prime }$ and $P^{\prime }$ in system (2) in right sides and consider

4 $$\begin{aligned} 0&=b-\frac{\beta _{1}S^*P^*}{1+\alpha _{1}P^*}-\frac{\beta _{2} S^*(I_{A}^*+I_{S}^*)}{1+\alpha _{2}(I_{A}^*+I_{S}^*)}+\varphi R^*-\mu S^*,\\ 0&=\frac{\beta _{1}S^*P^*}{1+\alpha _{1}P^*}+\frac{\beta _{2} S^*(I_{A}^*+I_{S}^*)}{1+\alpha _{2}(I_{A}^*+I_{S}^*)}-(\mu +\upsilon )E^*,\\ 0&=\eta \upsilon E^*-(\mu +\delta +\gamma _{S}+\tau _{S})I_{S}^*,\\ 0&=(1-\eta )\upsilon E^*-(\mu +\delta +\gamma _{A}+\tau _{A})I_{A}^*,\\ 0&=\gamma _{A}I_{A}^*+\gamma _{S}I_{S}^*-(\mu +\varphi ) R^*,\\ 0&=\tau _{A}I_{A}^*+\tau _{S}I_{S}^*-\mu _{P}P^*. \end{aligned}$$

From 3rd and 4th equations in the system (4), we get

$$\begin{aligned} I_{S}^*=\frac{\eta \upsilon }{\mu +\delta +\gamma _{S}+\tau _{S}}E^* \end{aligned}$$

and

$$\begin{aligned} I_{A}^*=\frac{(1-\eta )\upsilon }{\mu +\delta +\gamma _{A}+\tau _{A}}E^*. \end{aligned}$$

So

$$\begin{aligned} \tau _S I_{S}^*+\tau _A I_{A}^*=\left(\frac{\tau _S\eta \upsilon }{\mu +\delta +\gamma _{S}+\tau _{S}} +\frac{\tau _A(1-\eta )\upsilon }{\mu +\delta +\gamma _{A}+\tau _{A}}\right)E^*, \end{aligned}$$

or

$$\begin{aligned} \mu _{P}P^*=\left(\frac{\tau _S\eta \upsilon }{\mu +\delta +\gamma _{S}+\tau _{S}} +\frac{\tau _A(1-\eta )\upsilon }{\mu +\delta + \gamma _{A}+\tau _{A}}\right)E^*, \end{aligned}$$

or

$$\begin{aligned} P^*=\frac{1}{\mu _{P}}\left(\frac{\tau _S\eta \upsilon }{\mu +\delta +\gamma _{S}+\tau _{S}}+\frac{\tau _A(1-\eta )\upsilon }{\mu +\delta +\gamma _{A}+\tau _{A}}\right)E^*. \end{aligned}$$

For simplicity let

$$\begin{aligned}A=\frac{1}{\mu _{P}}\left(\frac{\tau _S\eta \upsilon }{\mu +\delta +\gamma _{S}+\tau _{S}} +\frac{\tau _A(1-\eta )\upsilon }{\mu +\delta +\gamma _{A}+\tau _{A}}\right), \end{aligned}$$

which implies that

$$\begin{aligned} P^*=AE^*. \end{aligned}$$

Furthermore

$$\begin{aligned}{} & {} \gamma _S I_{S}^*+\gamma _A I_{A}^*=\left(\frac{\gamma _S\eta \upsilon }{\mu +\delta +\gamma _{S}+\tau _{S}} +\frac{\gamma _A(1-\eta )\upsilon }{\mu +\delta +\gamma _{A}+\tau _{A}}\right)E^*,\\{} & {} (\mu +\varphi ) R^*=\left(\frac{\gamma _S\eta \upsilon }{\mu +\delta +\gamma _{S}+\tau _{S}} +\frac{\gamma _A(1-\eta )\upsilon }{\mu +\delta +\gamma _{A}+\tau _{A}}\right)E^*,\\{} & {} R^*=\frac{1}{\mu +\varphi }\left(\frac{\gamma _S\eta \upsilon }{\mu +\delta +\gamma _{S} +\tau _{S}}+\frac{\gamma _A(1-\eta )\upsilon }{\mu +\delta +\gamma _{A}+\tau _{A}}\right)E^*. \end{aligned}$$

Let

$$\begin{aligned} \omega =\frac{1}{\mu +\varphi }\left(\frac{\gamma _S\eta \upsilon }{\mu +\delta +\gamma _{S}+\tau _{S}}+\frac{\gamma _A(1-\eta )\upsilon }{\mu +\delta +\gamma _{A}+\tau _{A}}\right), \end{aligned}$$

then

$$\begin{aligned} R^*=\omega E^*. \end{aligned}$$

Furthermore

$$\begin{aligned} I_{S}^*+I_{A}^*=\left(\frac{\eta \upsilon }{\mu +\delta +\gamma _{S}+\tau _{S}} +\frac{(1-\eta )\upsilon }{\mu +\delta +\gamma _{A}+\tau _{A}}\right)E^*. \end{aligned}$$

For simplicity let

$$\begin{aligned} B=\left(\frac{\eta \upsilon }{\mu +\delta +\gamma _{S}+\tau _{S}}+\frac{(1-\eta )\upsilon }{\mu +\delta +\gamma _{A}+\tau _{A}}\right), \end{aligned}$$

then

$$\begin{aligned} I_{S}^*+I_{A}^*=BE^*. \end{aligned}$$

From second equation of system (2), we have

5 $$\begin{aligned} (ax-b)y+(cx-d)y^2=0. \end{aligned}$$

Adding first two equations of system (4) while using $R^*=\omega E^*$, we get

6 $$\begin{aligned} \mu x+(\mu +\upsilon -\varphi \omega )y=m, \end{aligned}$$

$$\begin{aligned} a&=\beta _{1}A+\beta _{2}B,\ b=(\mu +\upsilon )(\alpha _{1}A+\alpha _{2}B),\ c=(\beta _{1}\alpha _{2}+\alpha _{1}\beta _{2})AB,\\ d&=(\mu +\upsilon )\alpha _{1}\alpha _{2}AB,\ x=S^*,\ y=E^*,\ m=b,\\ A&=\frac{1}{\mu _{P}}\left(\frac{\tau _{S}\eta \upsilon }{\mu +\delta +\gamma _{S}+\tau _{S}} +\frac{\tau _{A}(1-\eta )\upsilon }{\mu +\delta +\gamma _{A}+\tau _{A}}\right),\\ B&=\left(\frac{\eta \upsilon }{\mu +\delta +\gamma _{S}+\tau _{S}}+\frac{(1-\eta )\upsilon }{\mu +\delta +\gamma _{A}+\tau _{A}}\right),\\ \omega&=\frac{1}{\mu +\varphi }\left(\frac{\gamma _S\eta \upsilon }{\mu +\delta +\gamma _{S}+\tau _{S}}+\frac{\gamma _A(1-\eta )\upsilon }{\mu +\delta +\gamma _{A}+\tau _{A}}\right). \end{aligned}$$

Solving equation (5) and (6), we get

7 $$\begin{aligned} (S^*, E^*, I_{S}^*, I_{A}^*, R^*, P^*)&=(\frac{b}{\mu }, 0, 0, 0, 0, 0). \end{aligned}$$

8 $$\begin{aligned} S^*&=\frac{mc+a(\mu +\upsilon )+d\mu +{\sqrt{(-cm+d\mu +a(\mu +\upsilon -\varphi \omega ))^2+4c(am-b\mu )(\mu +\upsilon -\varphi \omega )}-a\varphi \omega }}{2c\mu },\\ E^*&=\frac{mc-a(\mu +\upsilon )-d\mu -{\sqrt{(-cm+d\mu +a(\mu +\upsilon -\varphi \omega ))^2+4c(am-b\mu )(\mu +\upsilon -\varphi \omega )}+a\varphi \omega }}{2c(\mu +\upsilon -\varphi \omega )},\\ I_{S}^*&=\frac{\eta \upsilon \left\{mc-a(\mu +\upsilon )-d\mu -{\sqrt{(-cm+d\mu +a(\mu +\upsilon -\varphi \omega ))^2+4c(am-b\mu )(\mu +\upsilon -\varphi \omega )}+a\varphi \omega }\right\}}{2c(\mu +\upsilon -\varphi \omega )(\mu +\delta +\gamma _{S}+\tau _{S})},\\ I_{A}^*&=\frac{(1-\eta )\upsilon \left\{mc-a(\mu +\upsilon )-d\mu -{\sqrt{(-cm+d\mu +a(\mu +\upsilon -\varphi \omega ))^2+4c(am-b\mu )(\mu +\upsilon -\varphi \omega )}+a\varphi \omega }\right\}}{2c(\mu +\upsilon -\varphi \omega )(\mu +\delta +\gamma _{S}+\tau _{S})},\\ R^*&=\frac{\omega \left\{mc-a(\mu +\upsilon )-d\mu -{\sqrt{(-cm+d\mu +a(\mu +\upsilon -\varphi \omega ))^2+4c(am-b\mu )(\mu +\upsilon -\varphi \omega )}+a\varphi \omega }\right\}}{2c(\mu +\upsilon -\varphi \omega )},\\ P^*&=\frac{A\left\{mc-a(\mu +\upsilon )-d\mu -{\sqrt{(-cm+d\mu +a(\mu +\upsilon -\varphi \omega ))^2+4c(am-b\mu )(\mu +\upsilon -\varphi \omega )}+a\varphi \omega }\right\}}{2c(\mu +\upsilon -\varphi \omega )}. \end{aligned}$$

9 $$\begin{aligned} S^*&=\frac{mc+a(\mu +\upsilon )+d\mu -{\sqrt{(-cm+d\mu +a(\mu +\upsilon -\varphi \omega ))^2+4c(am-b\mu )(\mu +\upsilon -\varphi \omega )}-a\varphi \omega }}{2c\mu },\\ E^*&=\frac{mc-a(\mu +\upsilon )-d\mu +{\sqrt{(-cm+d\mu +a(\mu +\upsilon -\varphi \omega ))^2+4c(am-b\mu )(\mu +\upsilon -\varphi \omega )}+a\varphi \omega }}{2c(\mu +\upsilon -\varphi \omega )},\\ I_{S}^*&=\frac{\eta \upsilon \left\{mc-a(\mu +\upsilon )-d\mu +{\sqrt{(-cm+d\mu +a(\mu +\upsilon -\varphi \omega ))^2+4c(am-b\mu )(\mu +\upsilon -\varphi \omega )}+a\varphi \omega }\right\}}{2c(\mu +\upsilon -\varphi \omega )(\mu +\delta +\gamma _{S}+\tau _{S})},\\ I_{A}^*&=\frac{(1-\eta )\upsilon \left\{mc-a(\mu +\upsilon )-d\mu +{\sqrt{(-cm+d\mu +a(\mu +\upsilon -\varphi \omega ))^2+4c(am-b\mu )(\mu +\upsilon -\varphi \omega )}+a\varphi \omega }\right\}}{2c(\mu +\upsilon -\varphi \omega )(\mu +\delta +\gamma _{S}+\tau _{S})},\\ R^*&=\frac{\omega \left\{mc-a(\mu +\upsilon )-d\mu +{\sqrt{(-cm+d\mu +a(\mu +\upsilon -\varphi \omega ))^2+4c(am-b\mu )(\mu +\upsilon -\varphi \omega )}+a\varphi \omega }\right\}}{2c(\mu +\upsilon -\varphi \omega )},\\ P^*&=\frac{A\left\{mc-a(\mu +\upsilon )-d\mu +{\sqrt{(-cm+d\mu +a(\mu +\upsilon -\varphi \omega ))^2+4c(am-b\mu )(\mu +\upsilon -\varphi \omega )}+a\varphi \omega }\right\}}{2c(\mu +\upsilon -\varphi \omega )}. \end{aligned}$$

### Computation of $R_{0}$

The basic reproduction rate, often represented as $R_{0}$, quantifies the average count of subsequent infections initiated by one person within a population that is entirely susceptible. This metric serves as a gauge to determine if the infection is likely to propagate within the population. The calculation of $R_{0}$ employs the methodology known as the “next generation matrix approach”.

Since the first and fifth equations in system (2) are independent of each other, we have consequently reduced system (2) to the following:

10 $$\begin{aligned} E^{\prime }&=\frac{\beta _{1}SP}{1+\alpha _{1}P}+\frac{\beta _{2} S(I_{A}+I_{S})}{1+\alpha _{2}(I_{A}+I_{S})}-(\mu +\upsilon )E,\\ I^{\prime }_{S}&=\eta \upsilon E-(\mu +\delta +\gamma _{S}+\tau _{S})I_{S},\\ I^{\prime }_{A}&=(1-\eta )\upsilon E-(\mu +\delta +\gamma _{A}+\tau _{A})I_{A},\\ P^{\prime }&=\tau _{A}I_{A}+\tau _{S}I_{S}-\mu _{P}P. \end{aligned}$$

Let $x=(E,I_{A}, I_{S}, P)^{T},$ then the model can be written as $\frac{d}{dt}(x)=\mathcal{F}(x)-\mathcal{V}(x)$, where

$$\begin{aligned} \mathcal{F}(x)=\left( \begin{array}{c} \frac{\beta _{1}SP}{1+\alpha _{1}P}+\frac{\beta _{2} S(I_{A}+I_{S})}{1+\alpha _{2}(I_{A}+I_{S})}\\ 0\\ 0\\ \tau _{A}I_{A}+\tau _{S}I_{S} \end{array}\right) \quad \text{and}\quad \mathcal{V}(x)=\left( \begin{array}{c}(\mu +\upsilon )E\\ (\mu +\delta +\gamma _{S}+\tau _{S})I_{S}-\eta \upsilon E\\ (\mu +\delta +\gamma _{A}+\tau _{A})I_{A}-(1-\eta )\upsilon E\\ \mu _{P}P \end{array}\right) . \end{aligned}$$

Suppose *F* and *V* represent the Jacobian matrices of $\mathcal{F}$ and $\mathcal{V}$ at DFE respectively, then we obtain $FV^{-1}$ as

$$\begin{aligned} FV^{-1}=\left( \begin{array}{cccc} \frac{\beta _{2} b\eta \upsilon }{\mu c_{1}c_{2}}+\frac{\beta _{2} b(1-\eta )\upsilon }{\mu c_{1}c_{3}} &{} \frac{\beta _{2} b}{\mu c_{2}} &{} \frac{\beta _{2} b}{\mu c_{3}} &{} \frac{\beta _{1} b}{\mu \mu _{P}}\\ 0 &{} 0 &{} 0 &{} 0\\ 0 &{} 0 &{} 0 &{} 0\\ \frac{\tau _{S}\eta \upsilon }{c_{1}c_{2}}+\frac{\tau _{A}(1-\eta )\upsilon }{c_{1}c_{3}} &{} \frac{\tau _{S}}{c_{2}} &{} \frac{\tau _{A}}{c_{3}} &{} 0\end{array}\right) , \end{aligned}$$

where $c_{1}=\mu +\upsilon$, $c_{2}=\mu +\delta +\gamma _{S}+\tau _{S}$, and $c_{3}=\mu +\delta +\gamma _{A}+\tau _{A}$. The reproduction number $R_{0}$ is the largest eigenvalue of $FV^{-1}$, given by

$$\begin{aligned} R_{0}=\frac{\frac{\beta _{2}b\eta \upsilon }{\mu c_{1}c_{2}}+\frac{\beta _{2}b(1-\eta )\upsilon }{\mu c_{1}c_{3}}+\sqrt{(\frac{\beta _{2}b\eta \upsilon }{\mu c_{1}c_{2}}+\frac{\beta _{2}b(1-\eta )\upsilon }{\mu c_{1}c_{3}})^2+4\frac{\beta _{1} b}{\mu \mu _{P}}(\frac{\tau _{S}\eta \upsilon }{c_{1}c_{2}} +\frac{\tau _{A}(1-\eta )\upsilon }{c_{1}c_{3}})}}{2}. \end{aligned}$$

The notation stands for basic reproductive number for human $R_{0}^{H}$ and for pathogens $R_{0}^{P}$. Our remarks are recorded as:

11 $$\begin{aligned} R_{0}^{H}=\frac{\beta _{2}b}{\mu c_{1}}\left(\frac{\eta \upsilon }{c_{2}}+\frac{(1-\eta )\upsilon }{c_{3}}\right), \end{aligned}$$

and

12 $$\begin{aligned} R_{0}^{P}=\frac{\beta _{1}b}{c_{1}\mu \mu _{P}}\left(\frac{\tau _{S}\eta \upsilon }{c_{2}} +\frac{\tau _{A}(1-\eta )\upsilon }{c_{3}}\right), \end{aligned}$$

therefore

13 $$\begin{aligned} R_{0}=\frac{R_{0}^{H}+\sqrt{(R_{0}^{H})^{2}+4R_{0}^{P}}}{2}. \end{aligned}$$

**Figure 2:**
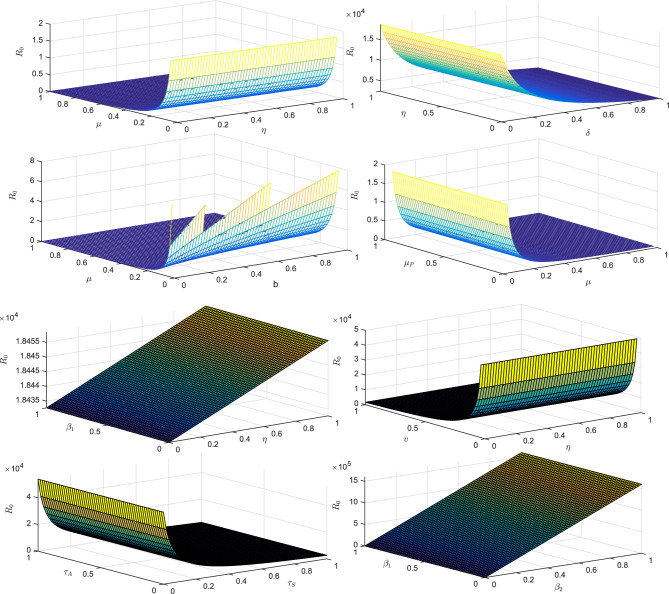
3D profile verses various parameters.

Here, $R_0$ is composed of two portions, which correspond to the two COVID-19 transmission modes. In addition, we present 3D profile of $R_0$ corresponding to various parameters to investigate the trends of parameters involve in (13) in Fig. 2 as follows:

## Local stability of DFE and EE

### Theorem 3.1

The DFE is locally asymptotically stable for $R_{0}<1$.

### Proof

The jacobian matrix with respect to the system (2) is given by

$$\begin{aligned} J_{0}=\left( \begin{array}{cccccc} -\mu &{} 0 &{} -\beta _{2}S^{0} &{} -\beta _{2}S^{0} &{} \varphi &{} 0\\ 0 &{} -(\mu +\upsilon ) &{} \beta _{2} S^{0} &{} \beta _{2} S^{0} &{} 0 &{} 0\\ 0 &{} \eta \upsilon &{} -(\mu +\delta +\gamma _{S}+\tau _{S}) &{} 0 &{} 0 &{} 0\\ 0 &{} (1-\eta )\upsilon &{} 0 &{} -(\mu +\delta +\gamma _{A}+\tau _{A}) &{} 0 &{} 0\\ 0 &{} 0 &{} \gamma _{S} &{} \gamma _{A} &{} -(\mu +\varphi ) &{} 0\\ 0 &{} 0 &{} \tau _{S} &{} \tau _{A} &{} 0 &{} -\mu _{P} \end{array}\right) \end{aligned}$$

The characteristic polynomial of $J_{0}$ about $(S^{0}, E^{0}, I_S^{0}, I_A^{0}, R^0, P^0)$ is given by

14 $$\begin{aligned} \mathbb{C}(\lambda )= & {} Det(J^0-\lambda I)\nonumber \\ \mathbb{C}(\lambda )= & {} (\lambda +\mu )\left(\lambda +(\mu +\varphi )\right)(\lambda +\mu _{P})\mathbb{Q}(\lambda ). \end{aligned}$$

Here

15 $$\begin{aligned} \mathbb{Q}(\lambda )=\lambda ^{3}+ a\lambda ^{2}+ b\lambda +c \end{aligned}$$

with

$$\begin{aligned}{}&a=(\mu +\upsilon )+(\delta +\mu +\gamma _{S}+\tau _{S})+(\delta +\mu +\gamma _{A}+\tau _{A}),\\&b=(\delta +\mu )+(\delta +3\mu +2\upsilon )+(\delta +2\mu +\upsilon ) (\tau _{S}+\tau _{A}+\gamma _{S}+\gamma _{A})+(\gamma _{S}+\gamma _{A}) \tau _{A}+\tau _{S}\tau _{A}+\gamma _{S}\gamma _{A} +\gamma _{A}\upsilon \beta _{2}S^{0},\\&c=(\mu +\upsilon )(\delta +\mu +\gamma _{A}+\tau _{A})(\delta +\mu +\gamma _{S} +\tau _{S})+S^0\upsilon \beta _{2}\eta \left((\gamma _{S}+\tau _{S}) -(\gamma _{A}+\tau _{A})\right)-S^{0}\upsilon \beta _{2}(\delta +\mu +\gamma _{S}+\tau _{S}). \end{aligned}$$

It follows from (14) that $\lambda _{1}=-\mu$, $\lambda _{2}=-(\mu +\varphi )$ and $\lambda _{3}=-\mu _P$ are the eigenvalues of $J_{0}$, its other eigenvalues are the roots of (15). Furthermore, it is clear that both $a>0$ and $b>0$. Considering the high sensitivity of parameters $\gamma _{S}$, $\tau _{S}$, and $\beta _{2}$, it is important to note that $(\gamma _{S}+\tau _{S})>(\gamma _{A}+\tau _{A})$ and $S^{0}\beta _{2}<(\delta +\mu +\gamma _{A}+\tau _{A})$ to maintain the local asymptotic stability of the DFE provided that $R_{0}<1$. Failure to meet these conditions would render the DFE locally asymptotically unstable provided that $R_{0}>1$. Consequently, we conclude that $c>0$. This analysis establishes that $ab-c>0$. This indicates that the real parts of the roots of (15) are negative. Thus, the system’s single positive equilibrium point of the system (2) is locally asymptotically stable according to the Hurwitz criterion. This implies that the DFE is locally asymptotically stable. This completes the proof. $\square$

### Local stability analysis of EE at $E_{*}$

In this subsection, we establish the asymptotic stability of (2) at $E_{*}$.

#### Theorem 3.2

The EE denoted by $E_{*}$ is locally asymptotically stable with $R_{0}>1$.

#### Proof

At $E_{*}$, the jacobian matrix of (2) can be deduced as

$$\begin{aligned} J_{*}=\left( \begin{array}{cccccc} -\frac{\beta _{1}P^*}{1+\alpha _{1}P^*}-\frac{\beta _{2}(I_{A}^*+I_{S}^*)}{1+\alpha _{2}(I_{A}^*+I_{S}^*)}-\mu &{} 0 &{} -\frac{\beta _{2}S^*}{(1+\alpha _{2}(I_{A}^*+I_{S}^*))^2} &{} -\frac{\beta _{2}S^*}{(1+\alpha _{2}(I_{A}^*+I_{S}^*))^2} &{} \varphi &{} -\frac{\beta _{1}S^*}{(1+\alpha _{1}P^*)^{2}}\\ \frac{\beta _{1}P^*}{1+\alpha _{1}P^*}+\frac{\beta _{2}(I_{A}^*+I_{S}^*)}{1+\alpha _{2}(I_{A}^*+I_{S}^*)} &{} -(\mu +\upsilon ) &{} \frac{\beta _{2}S^*}{(1+\alpha _{2}(I_{A}^*+I_{S}^*))^2} &{} \frac{\beta _{2}S^*}{(1+\alpha _{2}(I_{A}^*+I_{S}^*))^2} &{} 0 &{} \frac{\beta _{1}S^*}{(1+\alpha _{1}P^*)^{2}}\\ 0 &{} \eta \upsilon &{} -(\mu +\delta +\gamma _{S}+\tau _{S}) &{} 0 &{} 0 &{} 0\\ 0 &{} (1-\eta )\upsilon &{} 0 &{} -(\mu +\delta +\gamma _{A}+\tau _{S}) &{} 0 &{} 0\\ 0 &{} 0 &{} \gamma _{S} &{} \gamma _{A} &{} -(\mu +\varphi ) &{} 0\\ 0 &{} 0 &{} \tau _{S} &{} \tau _{A} &{} 0 &{} -\mu _{P} \end{array}\right) . \end{aligned}$$

For simplicity, let $k_{1}=\frac{\beta _{1}P^*}{1+\alpha _{1}P^*}+\frac{\beta _{2}(I_{A}^*+I_{S}^*)}{1+\alpha _{2}(I_{A}^*+I_{S}^*)}$, $k_{2}=\frac{\beta _{2}S^*}{(1+\alpha _{2}(I_{A}^*+I_{S}^*))^2}$, $k_{3}=\frac{\beta _{1}S^*}{(1+\alpha _{1}P^*)^{2}}$, $c_{1}=\mu +\upsilon$, $c_{2}=\mu +\delta +\gamma _{S}+\tau _{S}$ and $c_{3}=\mu +\delta +\gamma _{A}+\tau _{A}$. The matrix shown above can be expressed in the following manner:

$$\begin{aligned} J_{*}=\left( \begin{array}{cccccc} -k_{1}-\mu &{} 0 &{} -k_{2} &{} -k_{2} &{} \varphi &{} -k_{3}\\ k_{1} &{} -c_{1} &{} k_{2} &{} k_{2} &{} 0 &{} k_{3}\\ 0 &{} \eta \upsilon &{} -c_{2} &{} 0 &{} 0 &{} 0\\ 0 &{} (1-\eta )\upsilon &{} 0 &{} -c_{3} &{} 0 &{} 0\\ 0 &{} 0 &{} \gamma _{S} &{} \gamma _{A} &{} -(\mu +\varphi ) &{} 0\\ 0 &{} 0 &{} \tau _{S} &{} \tau _{A} &{} 0 &{} -\mu _{P} \end{array}\right) , \end{aligned}$$

here in this case

$$\begin{aligned} \textrm{Tr}(J_*)=-(k_{1}+2\mu +c_{1}+c_{2}+c_{3}+\varphi +\mu _{P})<0. \end{aligned}$$

We see that one eigenvalue of Jacobian matrix $J_{*}$ is clearly negative, i.e. $\lambda _{1}=-\mu _{P}<0$. The remaining eigenvalues can be tested by observing that $\det (J_{*})$ and $\textrm{Tr}(J_{*})$ have different signs. Now from the above jacobian matrix $J_{*}$, we have

$$\begin{aligned} \det (J_{*})=\left\{(\mu +\varphi )\left((\mu +k_{1})c_{1}c_{2}c_{3}+\upsilon \mu k_{2}\left(\eta (c_{2}-c_{3})-c_{2}\right)\right)+\varphi \upsilon k_{1}\left(\eta (c_{2}\gamma _{A}-c_{3}\gamma _{S})-c_{2}\gamma _{A}\right)\right\}\mu _{P}>0. \end{aligned}$$

Considering the high sensitivity of parameters $\eta$, $c_{2}$, and $c_{3}$, it is important to note that $\eta >\frac{c_{2}}{c_{2}-c_{3}}$ (assuming that $c_{2}\ne c_{3}$) to maintain the local asymptotic stability of the EE provided that $R_{0}>1$. Failure to meet these conditions would render the EE locally asymptotically unstable. In this context, we see that $\det (J_{*})$ and $\textrm{Tr}(J_{*})$ have opposite signs which insures that the eigenvalues of $J_{*}$ have negative real parts. This implies that the system (2) is locally asymptotically stable at $E_{*}$. $\square$

## Global stability of DFE and EE

In this section, we investigate the V–L stability of matrix *A* defined in (16) to determine the global stability of the endemic equilibrium $E_{*}$. The following definitions and preliminary lemmas are necessary prerequisites for this examination.

### Definition 4.1

^30,48–50^. Consider a square matrix, denoted as *M*, endowed with the property of symmetry and positive (negative) definiteness. In this context, the matrix *M* is succinctly expressed as $M>0$ or ($M<0$).

### Definition 4.2

^30,48–50^. We write a matrix $A_{n\times n}>0(A_{n\times n}<0)$ if $A_{n\times n}$ is symmetric positive (negative) definite.

### Definition 4.3

^30,48–50^. If a positive diagonal matrix $H_{n\times n}$ exists, such that $HA+A^{T}H^{T}<0$ then a non-singular matrix $A_{n\times n}$ is V–L stable.

### Definition 4.4

^30,48–50^. If a positive diagonal matrix $H_{n\times n}$ exists, such that $HA+A^{T}H^{T}<0$ $(>0)$ then, a non singular matrix $A_{n\times n}$ is diagonal stable.

The $2\times 2$ V–L stable matrix is determined by the following lemma.

### Lemma 4.1

^30,48–50^. Let $$B=\begin{bmatrix} B_{11} &{} B_{12} \\ B_{21} &{} B_{22}\\ \end{bmatrix}$$ is V–L stable if and only if $B_{11}<0$, $B_{22}<0$, and $\det (B)=B_{11}B_{22}-B_{12}B_{21}>0$.

### Lemma 4.2

^30,48–50^. Consider the nonsingular $A_{n\times n}=[A_{ij}]$, where $(n\ge 2)$, the positive diagonal matrix $C_{n\times n}=\textrm{diag}(C_{1},\ldots , C_{n})$, and $D=A^{-1}$ such that

$$\begin{aligned} {\left\{ \begin{array}{ll} A_{nn}>0,\\ \widetilde{C}\widetilde{A}+(\widetilde{C}\widetilde{A})^{T}>0,\\ \widetilde{C}\widetilde{D}+(\widetilde{C}\widetilde{D})^{T}>0. \end{array}\right. } \end{aligned}$$

Then, for $H_{n}>0$, it ensures that the matrix expression $HA + A^{T}H^{T}$ is positive definite.

### Global stability of DFE

We employ the same methodology used in^51^ to obtain the necessary outcomes for globally asymptotically.

#### Theorem 4.5

If $R_{0}<1$, the system (2) is globally asymptotically stable at ${E}_{0}=\left( \frac{b}{\sigma },0,0,0,0,0 \right)$.

#### Proof

Taking the Lyapunov function corresponding to all dependent classes of the model

$$\begin{aligned} L=m_1(S-S^*)+m_2(E-E^*)+m_3(I_S-I_S^*)+m_4(I_A-I_A^*)+m_5(R-R^*). \end{aligned}$$

Taking derivative with respect to *t* yields

$$\begin{aligned} L^\prime =m_1S^\prime +m_2E^\prime +m_3I_S^\prime +m_4I_A^\prime +m_5R^\prime . \end{aligned}$$

After utilizing the values derived from system (2) and performing requisite calculations, we obtain:

$$\begin{aligned} L ^\prime =& \,m_{1}b+(m_2-m_1)\frac{\beta _{1}SP}{1+\alpha _1P}+(m_2-m_1) \frac{\beta _2S(I_A+I_S)}{1+\alpha _2(I_A+I_S)}+(m_1-m_2)\varphi E+(m_4-m_2) \upsilon E+(m_3-m_4)\eta \upsilon E\\&+ (m_5-m_3)\gamma _S I_S+(m_5-m_4)\gamma _A I_A-m_1\mu S-m_2\mu E -m_3\mu I_S-m_3\delta I_S-m_4\mu I_A-m_4\delta I_A-m_5\mu R. \end{aligned}$$

Using $m_1=m_2=m_3=m_4=m_5=1$ in the above equation gives

$$\begin{aligned} L^\prime= & \, b-\mu S-\mu E-\mu I_S-\delta I_S-\mu I_A-\delta I_A-\mu R\\= & {} -\left(\mu N+\delta (I_{A}+I_{S})-b\right)<0. \end{aligned}$$

Thus, the system (2) is globally asymptotically stable at $E_{0}$, whenever $R_{0}<1$. $\square$

#### Global stability analysis of the EE at $E_{*}$

We propose the following Lyapunov function in order to prove the global stability at $E_{*}$.

$$\begin{aligned} L=m_1(S-S^*)^2+m_2(E-E^*)^2+m_3(I_S-I_S^*)^2+m_4(I_A-I_A^*)^2+m_5(R-R^*)^2+m_6(P-P^*)^2. \end{aligned}$$

Here, $m_{1}$, $m_{2}$, $m_{3}$, $w_{4}$, $m_{5}$, and $w_{6}$ are non-negative constants. By taking the derivative of *L* with respect to time along the trajectories of the model (8), one has

$$\begin{aligned} L^\prime =2m_1(S-S^*)S^\prime +2m_2(E-E^*)E^\prime +2m_3(I_S-I_S^*)I_S^\prime +2m_4(I_A-I_A^*)I_A^\prime +2m_5(R-R^*)R^\prime +2m_6(P-P^*)P^\prime . \end{aligned}$$

Here

$$\begin{aligned}&2m_1(S-S^*)S^\prime \\&\quad =2m_1(S-S^*)\bigg\{-\frac{\beta _{1}SP}{1+\alpha _{1}P}-\frac{\beta _{2}S(I_A+I_S)}{1+\alpha _{2}(I_A+I_S)}+\frac{\beta _{1}S^*P^*}{1+\alpha _{1}P^*}+ \frac{\beta _{2}S^*(I_A^*+I_S^*)}{1+\alpha _{2}(I_A^*+I_S^*)}+\varphi (R-R^*)-\mu (S-S^*)\bigg\}\\&\quad =2m_1(S-S^*)\bigg\{-\bigg(\frac{\beta _{1}P}{1+\alpha _{1}P}+\frac{\beta _{2}(I_A+I_S)}{1+\alpha _{2}(I_A+I_S)}\bigg)S+\bigg(\frac{\beta _{1}P^*}{1+\alpha _{1}P^*}+ \frac{\beta _{2}(I_A^*+I_S^*)}{1+\alpha _{2}(I_A^*+I_S^*)}\bigg)S^*+\varphi (R-R^*)-\mu (S-S^*)\bigg\}\\&\quad =2m_1(S-S^*)\bigg\{-\textbf{A}S+\textbf{B}S^*+\varphi (R-R^*)-\mu (S-S^*)\bigg\}\\&\quad =2m_1(S-S^*)\bigg\{-\textbf{A}S+\textbf{A}S^*-\textbf{A}S^*+\textbf{B}S^*+\varphi (R-R^*)-\mu (S-S^*)\bigg\}\\&\quad =2m_1(S-S^*)\bigg\{-\textbf{A}(S-S^*)-(\textbf{A}-\textbf{B})S^*+\varphi (R-R^*)-\mu (S-S^*)\bigg\}\\&\quad =2m_1(S-S^*)\bigg\{-(\textbf{A}+\mu )(S-S^*)-(\textbf{A}-\textbf{B})S^*+\varphi (E-E^*)\bigg\}\\&\quad =2m_1(S-S^*)\bigg\{-\bigg(\frac{\beta _{1}P}{1+\alpha _{1}P}+\frac{\beta _{2}(I_A+I_S)}{1+\alpha _{2}(I_A+I_S)}+\mu \bigg)(S-S^*) -\frac{\beta _{1}S^*}{(1+\alpha _{1}P)(1+\alpha _{1}P^*)}(P-P^*)\\&\qquad -\frac{\beta _{2}S^*}{(1+\alpha _{2}(I_A+I_S))(1+\alpha _{2}(I_A^*+I_S^*))}(I_A-I_A^*) -\frac{\beta _{2}S^*}{(1+\alpha _{2}(I_A+I_S))(1+\alpha _{2}(I_A^*+I_S^*))}(I_S-I_S^*)+\varphi (R-R^*)\bigg\}\\&\quad =2m_1(S-S^*)\bigg\{-\bigg(\frac{\beta _{1}P}{1+\alpha _{1}P}+\frac{\beta _{2}(I_A+I_S)}{1+\alpha _{2}(I_A+I_S)}+\mu \bigg)(S-S^*)+\varphi (R-R^*)\\&\qquad -\frac{\beta _{2}S^*}{(1+\alpha _{2}(I_A+I_S))(1+\alpha _{2}(I_A^*+I_S^*))}(I_S-I_S^*)-\frac{\beta _{2}S^*}{(1+\alpha _{2}(I_A+I_S))(1+\alpha _{2}(I_A^*+I_S^*))}(I_A-I_A^*)\\&\qquad -\frac{\beta _{1}S^*}{(1+\alpha _{1}P)(1+\alpha _{1}P^*)}(P-P^*)\bigg\},\\ \end{aligned}$$

$$\begin{aligned}&2m_2(E-E^*)E^\prime \\&\quad =2m_2(E-E^*)\bigg\{\bigg(\frac{\beta _{1}P}{1+\alpha _{1}P}+\frac{\beta _{2}(I_A+I_S)}{1+\alpha _{2}(I_A+I_S)}\bigg)S-\bigg(\frac{\beta _{1}P^*}{1+\alpha _{1}P^*}+ \frac{\beta _{2}(I_A^*+I_S^*)}{1+\alpha _{2}(I_A^*+I_S^*)}\bigg)S^*-(\mu +\upsilon )(E-E^*)\bigg\}\\&\quad =2m_2(E-E^*)\bigg\{\textbf{A}S-\textbf{B}S^*-(\mu +\upsilon )(E-E^*)\bigg\}\\&\quad =2m_2(E-E^*)\bigg\{\textbf{A}S-\textbf{A}S^*+\textbf{A}S^*-\textbf{B}S^*-(\mu +\upsilon )(E-E^*)\bigg\}\\&\quad =2m_2(E-E^*)\bigg\{\textbf{A}(S-S^*)+(\textbf{A}-\textbf{B})S^*-(\mu +\upsilon )(E-E^*)\bigg\}\\&\quad =2m_2(E-E^*)\bigg\{\bigg(\frac{\beta _{1}P}{1+\alpha _{1}P}+\frac{\beta _{2}(I_A+I_S)}{1+\alpha _{2}(I_A+I_S)}\bigg)(S-S^*)+ \frac{\beta _{1}S^*}{(1+\alpha _{1}P)(1+\alpha _{1}P^*)}(P-P^*)\\&\qquad +\frac{\beta _{2}S^*}{(1+\alpha _{2}(I_A+I_S))(1+\alpha _{2}(I_A^*+I_S^*))}(I_A-I_A^*) +\frac{\beta _{2}S^*}{(1+\alpha _{2}(I_A+I_S))(1+\alpha _{2}(I_A^*+I_S^*))}(I_S-I_S^*)-(\mu +\upsilon )(E-E^*)\bigg\}\\&\quad =2m_2(E-E^*)\bigg\{\bigg(\frac{\beta _{1}P}{1+\alpha _{1}P}+\frac{\beta _{2}(I_A+I_S)}{1+\alpha _{2}(I_A+I_S)}\bigg)(S-S^*)-(\mu +\upsilon )(E-E^*)\\&\qquad +\frac{\beta _{2}S^*}{(1+\alpha _{2}(I_A+I_S))(1+\alpha _{2}(I_A^*+I_S^*))}(I_S-I_S^*)+\frac{\beta _{2}S^*}{(1+\alpha _{2}(I_A+I_S))(1+\alpha _{2}(I_A^*+I_S^*))}(I_A-I_A^*)\\&\qquad +\frac{\beta _{1}S^*}{(1+\alpha _{1}P)(1+\alpha _{1}P^*)}(P-P^*)\bigg\},\\&2m_3(I_S-I_S^*)I_S^\prime =2m_3(I_S-I_S^*)\bigg\{\eta \upsilon (E-E^*)-(\mu +\delta +\gamma _S+\tau _{S})(I_S-I_S^*)\bigg\},\\&2m_4(I_A-I_A^*)I_A^\prime =2m_4(I_A-I_A^*)\bigg\{(1-\eta )\upsilon (E-E^*)-(\mu +\delta +\gamma _A+\tau _{A})(I_A-I_A^*)\bigg\},\\&2m_5(R-R^*)R^\prime =2m_5(R-R^*)\bigg\{\gamma _A(I_A-I_A^*)+\gamma _S(I_S-I_S^*)-(\mu +\varphi )(R-R^*)\bigg\}, \quad \textrm{and}\\&2m_6(P-P^*)P^\prime =2m_6(P-P^*)\bigg\{\tau _A(I_A-I_A^*)+\tau _S(I_S-I_S^*)-\mu _P(P-P^*)\bigg\}. \end{aligned}$$

Using these values in $L^\prime$, which implies that $L^\prime =Z(MA+A^{T}M)Z^{T}.$

Here, $$Z=[S-S^*, E-E^*, I_S-I_S^*, I_A-I_A^*, R-R^*, P-P^*]$$, $$M={\textbf{diag}}(m_1, m_2, m_3, m_4, m_5, m_6)$$, and

16 $$\begin{aligned} A= & {} \left( \begin{array}{ccc} -\frac{\beta _{1}P}{1+\alpha _{1}P}-\frac{\beta _{2}(I_A+I_S)}{1+\alpha _{2}(I_A+I_S)}-\mu &{} 0 &{} -\frac{\beta _{2}S^*}{(1+\alpha _{2}(I_A+I_S))(1+\alpha _{2}(I_A^*+I_S^*))}\\ \frac{\beta _{1}P}{1+\alpha _{1}P}+\frac{\beta _{2}(I_A+I_S)}{1+\alpha _{2}(I_A+I_S)} &{} -(\mu +\upsilon ) &{} \frac{\beta _{2}S^*}{(1+\alpha _{2}(I_A+I_S))(1+\alpha _{2}(I_A^*+I_S^*))}\\ 0 &{} \eta \upsilon &{} -(\mu +\delta +\gamma _S+\tau _{S})\\ 0 &{} (1-\eta )\upsilon &{} 0\\ 0 &{} 0 &{} \gamma _S\\ 0 &{} 0 &{} \tau _S\\ \end{array}\right. \nonumber \\{} & {} \left. \begin{array}{ccc} -\frac{\beta _{2}S^*}{(1+\alpha _{2}(I_A+I_S))(1+\alpha _{2}(I_A^*+I_S^*))} &{} \varphi &{} -\frac{\beta _{1}S^*}{(1+\alpha _{1}P)(1+\alpha _{1}P^*)}\\ \frac{\beta _{2}S^*}{(1+\alpha _{2}(I_A+I_S))(1+\alpha _{2}(I_A^*+I_S^*))} &{} 0 &{} \frac{\beta _{1}S^*}{(1+\alpha _{1}P)(1+\alpha _{1}P^*)}\\ 0 &{} 0 &{} 0\\ -(\mu +\delta +\gamma _A+\tau _{A}) &{} 0 &{} 0\\ \gamma _A &{} -(\mu +\varphi ) &{} 0\\ \tau _A &{} 0 &{} -\mu _P \end{array}\right) . \end{aligned}$$

It is important to note that matrix $A_{(n-1)\times (n-1)}$ is derived from matrix *A* by removing its final row and column. In accordance with the works of Zahedi and Kargar^48^, Shao and Shateyi^49^, Masoumnezhad et al.^50^, Chien and Shateyi^30^, and Parsaei et al.^29^, we introduce a series of lemmas and theorems to examine the global stability of the EE at $E_{*}$.

In order to make the calculation easier, we present the matrix (16) in more simplified form as given below:

17 $$\begin{aligned} A=\left( \begin{array}{cccccc} -a &{} 0 &{} -b &{} -b &{} c &{} -d\\ e &{} -f &{} b &{} b &{} 0 &{} d\\ 0 &{} g &{} -h &{} 0 &{} 0 &{} 0\\ 0 &{} i &{} 0 &{} -j &{} 0 &{} 0\\ 0 &{} 0 &{} k &{} l &{} -m &{} 0\\ 0 &{} 0 &{} n &{} p &{} 0 &{} -q\\ \end{array}\right) . \end{aligned}$$

Here, $$a=\frac{\beta _{1}P}{1+\alpha _{1}P}+\frac{\beta _{2}(I_A+I_S)}{1+\alpha _{2}(I_A+I_S)}+\mu$$, $$b=\frac{\beta _{2}S^*}{(1+\alpha _{2}(I_A+I_S))(1+\alpha _{2}(I_A^*+I_S^*))}$$, $$c=\varphi$$, $$d=\frac{\beta _{1}S^*}{(1+\alpha _{1}P)(1+\alpha _{1}P^*)}$$, $$e=\frac{\beta _{1}P}{1+\alpha _{1}P}+\frac{\beta _{2}(I_A+I_S)}{1+\alpha _{2}(I_A+I_S)}$$, $$f=(\mu +\upsilon )$$, $$g=\eta \upsilon$$, $$h=(\mu +\delta +\gamma _S+\tau _{S})$$, $$i=(1-\eta )\upsilon$$, $$j=(\mu +\delta +\gamma _A+\tau _{A})$$, $$k=\gamma _S$$, $$l=\gamma _A$$, $$m=(\mu +\varphi )$$, $$n=\tau _S$$, $$p=\tau _A$$, and $$q=\mu _P$$.

It is important to note that all the parameters *a*, *b*, *c*, *d*, *e*, *f*, *g*, *h*, *i*, *j*, *k*, *l*, *m*, *n*, *p*, and *q* are positive. Throughout this paper, it is important to note that $a-e$ and $-a+e$ are always equal to $\mu$ and $-\mu$ respectively. Moreover, we conclude that all the diagonal elements (the negative values) of the matrix *A* are negative, which is a good sign for stability. We need to compute the eigenvalues to make a definitive conclusion for the global stability of $E_{*}$. But finding the eigenvalues of the matrix *A* is quite time consuming. Therefore, we introduce a series of lemmas and theorems given below to examine whether all the eigenvalues of the matrix *A* have negative real parts or not?.

##### Theorem 4.6

The matrix $A_{6\times 6}$ defined in (17) is V–L stable.

##### Proof

Obviously $-A_{66}>0$. Consider $C=-\widetilde{A}$ be a $5\times 5$ matrix that is produced by eliminating the final row and column $-A$. Utilizing Lemma 4.2, we can demonstrate the diagonal stability of matrices $C=-\widetilde{A}$ and $\mathbb{C}=-\widetilde{A^{-1}}$. Let examine the matrix $C=-\widetilde{A}$ and $\mathbb{C}=-\widetilde{A^{-1}}$, from (17) we obtain

$$\begin{aligned} C=\left( \begin{array}{ccccc} a &{} 0 &{} b &{} b &{} -c\\ -e &{} f &{} -b &{} -b &{} 0\\ 0 &{} -g &{} h &{} 0 &{} 0\\ 0 &{} -i &{} 0 &{} j &{} 0\\ 0 &{} 0 &{} -k &{} -l &{} m \end{array}\right) , \quad \textrm{and}\quad \,\mathbb{C}=\frac{1}{\det (-A)}\left( \begin{array}{ccccc} \mathbf{{c}_{11}} &{} \mathbf{c_{12}} &{} \mathbf{c_{13}} &{} \mathbf{c_{14}} &{} \mathbf{c_{15}}\\ \mathbf{{c}_{21}} &{} \mathbf{c_{22}} &{} \mathbf{c_{23}} &{} \mathbf{c_{24}} &{} \mathbf{c_{25}}\\ \mathbf{{c}_{31}} &{} \mathbf{c_{32}} &{} \mathbf{c_{33}} &{} \mathbf{c_{34}} &{} \mathbf{c_{35}}\\ \mathbf{{c}_{41}} &{} \mathbf{c_{42}} &{} \mathbf{c_{43}} &{} \mathbf{c_{44}} &{} \mathbf{c_{45}}\\ \mathbf{{c}_{51}} &{} \mathbf{c_{52}} &{} \mathbf{c_{53}} &{} \mathbf{c_{54}} &{} \mathbf{c_{55}}\\ \end{array}\right) . \end{aligned}$$

Here

$$\begin{aligned} \det (-A)=-d (a - e) m (g j n +h i p) - \left(c e (g j k +h i l) + (b (a - e) h i + (b (a - e) g - a f h) j) m\right) q<0, \end{aligned}$$

and

$$\begin{aligned} \mathbf{c_{11}}= & {} -m\left(d g j n + d h i p + b h i q + b g j q - f h j q\right),\\ \mathbf{c_{12}}= & {} -d m (g j n + h i p) + \left(c (g j k + h i l) - b (h i + g j) m\right) q,\\ \mathbf{c_{13}}= & {} -f j m (d n + b q) + c \left(d i (l n - k p) + (f j k + b i (-k + l)) q\right),\\ \mathbf{c_{14}}= & {} -f h m (d p + b q) + c \left(d g (-l n + k p) + (b g (k - l) + f h l) q\right),\\ \mathbf{c_{15}}= & {} -c \left(d g j n + d h i p + b h i q + b g j q - f h j q\right),\\ \mathbf{c_{21}}= & {} e h j m q, \quad \mathbf{c_{22}}=a h j m q,\\ \mathbf{c_{23}}= & {} d (a - e) j m n + j \left(c e k + b (a - e) m\right) q,\\ \mathbf{c_{24}}= & {} d (a - e) h m p + h \left(c e l + b (a - e) m\right) q,\\ \mathbf{c_{25}}= & {} c e h j q,\quad \mathbf{c_{31}}=e g j m q,\\ \mathbf{c_{32}}= & {} a g j m q,\\ \mathbf{c_{33}}= & {} -a m \left(d i p + b i q - f j q\right) + e i \left(d m p - c l q + b m q\right),\\ \mathbf{c_{34}}= & {} d (a - e) g m p + g \left(c e l + b (a - e) m\right) q,\\ \mathbf{c_{35}}= & {} c e g j q,\quad \mathbf{c_{41}}=e h i m q,\quad \mathbf{c_{42}}=a h i m q,\\ \mathbf{c_{43}}= & {} d (a - e) i m n + i \left(c e k + b (a - e) m\right) q,\\ \mathbf{c_{44}}= & {} -a m \left(d g n + b g q - f h q\right) + e g \left(d m n - c k q + b m q\right),\\ \mathbf{c_{45}}= & {} c e h i q,\\ \mathbf{c_{51}}= & {} e \left(g j k + h i l\right) q,\\ \mathbf{c_{52}}= & {} a \left(g j k + h i l\right) q, \\ \mathbf{c_{53}}= & {} d (a - e) i (l n - k p) + \left(b e i (k - l) + a (f j k + b i (-k + l))\right) q,\\ \mathbf{c_{54}}= & {} d (-a + e) g (l n - k p) + \left(b e g (-k + l) + a (b g (k - l) + f h l)\right) q,\\ \mathbf{c_{55}}= & {} d (-a + e) (g j n + h i p) + \left(b (-a + e) h i + \left(-a b g + b e g + a f h\right) j\right) q. \end{aligned}$$

Since

$$\begin{aligned} \det (-A)=-d (a - e) m (g j n +h i p) - \left(c e (g j k +h i l) + \left(b (a - e) h i + (b (a - e) g - a f h) j\right) m\right) q<0, \end{aligned}$$

and

$$\begin{aligned} \mathbf{c_{55}}=d (-a + e) (g j n + h i p) + \left(b (-a + e) h i + ((-a + e)b g + a f h) j\right) q. \end{aligned}$$

Therefore

$$\begin{aligned} \mathbb{C}_{55}=\frac{-\left(\mu (d g j n + d h i p + b h i q + b g j q) - a f h j q\right)}{-\mu m (d g j n + d h i p + b h i q + b g j q)-(c e (g j k + h i l) - a f h j m) q}, \end{aligned}$$

which implies that

$$\begin{aligned} \mathbb{C}_{55}=\frac{\mu \left(d g j n + d h i p + b h i q + b g j q\right) - a f h j q}{\mu m \left(d g j n + d h i p + b h i q + b g j q\right)+\left(c e (g j k + h i l) - a f h j m\right) q}>0. \end{aligned}$$

Using the information provided in Lemma 4.2, we assert and demonstrate that $C=-\widetilde{A}$ and $\mathbb{C}=-\widetilde{A^{-1}}$ exhibit diagonal stability, thereby establishing the V–L stability of the matrix *A*. $\square$

##### Lemma 4.3

In accordance with Theorem 4.6, the matrix $C=-\widetilde{A}$ is affirmed to possess diagonal stability.

##### Proof

As $C_{55}>0$, using Lemma 4.2, we must demonstrate the diagonal stability of the reduced matrix $D=\widetilde{C}$ and its inverse $\mathbb{D}=\widetilde{C^{-1}}$ to complete the proof. Thus the matrix *D* can be obtained from the matrix *C* given in Theorem 4.6, we have

$$\begin{aligned} D=\left( \begin{array}{cccc} a &{} 0 &{} b &{} b\\ -e &{} f &{} -b &{} -b\\ 0 &{} -g &{} h &{} 0\\ 0 &{} -i &{} 0 &{} j \end{array}\right) , \quad \textrm{and}\quad \,\,\mathbb{D}=\frac{1}{\det (C)}\left( \begin{array}{cccc} \mathbf{d_{11}} &{} \mathbf{d_{12}} &{} \mathbf{d_{13}} &{} \mathbf{d_{14}}\\ \mathbf{d_{21}} &{} \mathbf{d_{22}} &{} \mathbf{d_{23}} &{} \mathbf{d_{24}}\\ \mathbf{d_{31}} &{} \mathbf{d_{32}} &{} \mathbf{d_{33}} &{} \mathbf{d_{34}}\\ \mathbf{d_{41}} &{} \mathbf{d_{42}} &{} \mathbf{d_{43}} &{} \mathbf{d_{44}} \end{array}\right) , \end{aligned}$$

here

$$\begin{aligned} \det (C)=-c e (g j k + h i l) + \left(b (-a + e) h i + ((-a + e)b g + a f h) j\right) m<0, \end{aligned}$$

and

$$\begin{aligned} \mathbf{d_{11}}= & {} -(b h i + b g j - f h j) m,\\ \mathbf{d_{12}}= & {} c (g j k + h i l) - b (h i + g j) m,\\ \mathbf{d_{13}}= & {} c f j k - b \left(c i (k - l) + f j m\right),\ \ \\ \mathbf{d_{14}}= & {} b c g (k - l) + f h (c l - b m),\\ \mathbf{d_{21}}= & {} e h j m,\ \ \mathbf{d_{22}}=a h j m,\\ \mathbf{d_{23}}= & {} j \left(c e k + b (a - e) m\right),\\ \mathbf{d_{24}}= & {} h \left(c e l + b (a - e) m\right),\\ \mathbf{d_{31}}= & {} e g j m,\ \ \mathbf{d_{32}}=a g j m,\\ \mathbf{d_{33}}= & {} -c e i l + \left((-a + e)b i + a f j\right) m,\\ \mathbf{d_{34}}= & {} g \left(c e l + b (a - e) m\right),\\ \mathbf{d_{41}}= & {} e h i m,\ \ \mathbf{d_{42}}=a h i m,\\ \mathbf{d_{43}}= & {} i \left(c e k + b (a - e) m\right),\\ \mathbf{d_{44}}= & {} -c e g k + \left((-a + e)b g + a f h\right) m. \end{aligned}$$

Since

$$\begin{aligned} \det (C)=-c e (g j k + h i l) + \left(b (-a + e) h i + \left((-a + e) b g + a f h\right) j\right) m, \end{aligned}$$

and

$$\begin{aligned} \mathbf{d_{44}}=-c e g k + \left((-a + e)b g + a f h\right) m. \end{aligned}$$

Therefore

$$\begin{aligned} \mathbb{D}_{44}=\frac{c e g k +\mu b g m - a f h m}{ce(gjk+hil)+b\mu (hi+gj)m-afhjm}>0. \end{aligned}$$

$$\square$$

##### Lemma 4.4

The matrix $\mathbb{C}=-\widetilde{A^{-1}}$ defined in Theorem 4.6 is diagonal stable.

##### Proof

Consider the matrix $\mathbb{C}$ in 4.6, we see that $\mathbb{C}_{55}>0$, Using Lemma 4.2, we must demonstrate that the modified matrix $E=\widetilde{\mathbb{C}}$ and its inverse $\mathbb{E}=\widetilde{\mathbb{C}^{-1}}$ exhibit diagonal stability. This establishes the completion of the proof. Thus the matrix *D* can be obtained from the matrix $\mathbb{C}$ given in Theorem 4.6, we have

$$\begin{aligned} E=\frac{1}{\det (-A)}\left( \begin{array}{cccc} \mathbf{{c}_{11}} &{} \mathbf{c_{12}} &{} \mathbf{c_{13}} &{} \mathbf{c_{14}}\\ \mathbf{{c}_{21}} &{} \mathbf{c_{22}} &{} \mathbf{c_{23}} &{} \mathbf{c_{24}}\\ \mathbf{{c}_{31}} &{} \mathbf{c_{32}} &{} \mathbf{c_{33}} &{} \mathbf{c_{34}}\\ \mathbf{{c}_{41}} &{} \mathbf{c_{42}} &{} \mathbf{c_{43}} &{} \mathbf{c_{44}}\\ \end{array}\right) ,\,\quad \textrm{and}\quad \,\mathbb{E}=\frac{1}{\det (\mathbb{C})}\left( \begin{array}{cccc} \mathbf{{e}_{11}} &{} \mathbf{e_{12}} &{} \mathbf{e_{13}} &{} \mathbf{e_{14}}\\ \mathbf{{e}_{21}} &{} \mathbf{e_{22}} &{} \mathbf{e_{23}} &{} \mathbf{e_{24}}\\ \mathbf{{e}_{31}} &{} \mathbf{e_{32}} &{} \mathbf{e_{33}} &{} \mathbf{e_{34}}\\ \mathbf{{e}_{41}} &{} \mathbf{e_{42}} &{} \mathbf{e_{43}} &{} \mathbf{e_{44}}\\ \end{array}\right) . \end{aligned}$$

Here

$$\begin{aligned} \det (\mathbb{C})&=\frac{q\ell ^{4}}{\det (-A)}<0, \end{aligned}$$

and

$$\begin{aligned} \mathbf{e_{11}}= & {} -a q\ell ^{3},\ \ \mathbf{e_{12}}=0,\ \ \mathbf{e_{13}}=-(d n + b q)\ell ^{3},\\ \mathbf{e_{14}}= & {} -(d p + b q)\ell ^{3},\ \ \mathbf{e_{21}}=e q\ell ^{3},\ \ \mathbf{e_{22}}=-f q\ell ^{3},\\ \mathbf{e_{23}}= & {} (d n + b q)\ell ^{3},\ \ \mathbf{e_{24}}=(d p + b q)\ell ^{3},\ \ \mathbf{e_{31}}=0,\\ \mathbf{e_{32}}= & {} g q \ell ^{3},\ \ \mathbf{e_{33}}=-h q\ell ^{3},\ \ \mathbf{e_{34}}=0,\ \ \mathbf{e_{41}}=0,\\ \mathbf{e_{42}}= & {} i q\ell ^{3},\ \ \mathbf{e_{43}}=0,\ \ \mathbf{e_{44}}=-j q\ell ^{3}. \end{aligned}$$

Here

$$\begin{aligned} \ell =\left[d (a - e) m (g j n + h i p) + \left\{c e (g j k + h i l) + \left(b (a - e) h i + (b (a - e) g - a f h) j\right) m\right\} q\right] \end{aligned}$$

Since

$$\begin{aligned} \det (\mathbb{C})&=\frac{q\ell ^{4}}{\det (-A)}, \end{aligned}$$

and

$$\begin{aligned} \mathbf{e_{44}}=-j q \ell ^{3}. \end{aligned}$$

Therefore

$$\begin{aligned} \mathbb{E}_{44}=\frac{-j}{\det (-A)\ell }>0, \end{aligned}$$

where $$\det (-A)<0$$ and $$\ell >0$$. $$\square$$

##### Lemma 4.5

The matrix *D*, as described in Lemma 4.3 and denoted by $\widetilde{C}$, exhibits diagonal stability.

##### Proof

It is clear that $D_{44}>0$. By applying Lemma 4.2, our task is to demonstrate the diagonal stability of the reduced matrix $F=\widetilde{D}$ and its inverse $\mathbb{F}=\widetilde{D^{-1}}$, thereby completing the proof. Thus from Lemma 4.3, we have

$$\begin{aligned} F=\left( \begin{array}{ccc} a &{} 0 &{} b\\ -e &{} f &{} -b\\ 0 &{} -g &{} h \end{array}\right) , \quad \textrm{and}\quad \,\,\mathbb{F}=\frac{1}{\det ({D})}\left( \begin{array}{ccc} \mathbf{f_{11}} &{} \mathbf{f_{12}} &{} \mathbf{f_{13}}\\ \mathbf{f_{21}} &{} \mathbf{f_{22}} &{} \mathbf{f_{23}}\\ \mathbf{f_{31}} &{} \mathbf{f_{32}} &{} \mathbf{f_{33}} \end{array}\right) . \end{aligned}$$

Here

$$\begin{aligned} \det (D)=b (-a + e) h i + \left((-a + e)b g + a f h\right) j>0 \ \ (\textrm{see}\ \ \textrm{Proposition}\ \ 4.1\ \ (iii)),\ \ \end{aligned}$$

and $$\mathbf{f_{11}}=f h j - b (h i + g j)>0 \ \ (\textrm{see}\ \ \textrm{Proposition}\ \ 4.1\ \ (iv))$$, $$\mathbf{f_{12}}=-b (h i + g j)$$, $$\mathbf{f_{13}}=b f j$$, $$\mathbf{f_{21}}=e h j$$, $$\mathbf{f_{22}}=a h j$$, $$\mathbf{f_{23}}=b (a - e) j$$, $$\mathbf{f_{31}}=e g j$$, $$\mathbf{f_{32}}=a g j$$, $$\mathbf{f_{33}}=(-a + e) b i + a f j>0 \ \ (\textrm{see}\ \ \textrm{Proposition}\ \ 4.1\ \ (v))$$.

Since $\det (D)>0$, and $\mathbf{f_{33}}>0$, therefore $\mathbb{F}_{33}>0$. $\square$

##### Lemma 4.6

The matrix $\mathbb{D}$, as defined in Lemma 4.3 denoted by $\widetilde{C^{-1}}$, exhibits diagonal stability.

##### Proof

It is obvious that $\mathbb{D}_{44}>0$, by employing Lemma 4.2, our task is to demonstrate the diagonal stability of a modified matrix $G=\widetilde{\mathbb{D}}$ and its inverse $\mathbb{G}=\widetilde{\mathbb{D}^{-1}}$. This verification serves as the completion of the proof. Thus from Lemma 4.3, we have

$$\begin{aligned} G=\frac{1}{\det (C)}\left( \begin{array}{ccc} \mathbf{d_{11}} &{} \mathbf{d_{12}} &{} \mathbf{d_{13}}\\ \mathbf{d_{21}} &{} \mathbf{d_{22}} &{} \mathbf{d_{23}}\\ \mathbf{d_{31}} &{} \mathbf{d_{32}} &{} \mathbf{d_{33}} \end{array}\right) , \quad \textrm{and}\quad \,\mathbb{G}=\frac{1}{\det (\mathbb{D})}\left( \begin{array}{ccc} \mathbf{g_{11}} &{} \mathbf{g_{12}} &{} \mathbf{g_{13}}\\ \mathbf{g_{21}} &{} \mathbf{g_{22}} &{} \mathbf{g_{23}}\\ \mathbf{g_{31}} &{} \mathbf{g_{32}} &{} \mathbf{g_{33}} \end{array}\right) . \end{aligned}$$

Here

$$\begin{aligned} \det (\mathbb{D})&=\frac{-m\ell _{1}^{3}}{\det (C)}>0, \end{aligned}$$

and

$$\begin{aligned} \mathbf{g_{11}}= & a m\ell _{1}^{2},\ \ \mathbf{g_{12}}=0,\ \ \mathbf{g_{13}}=-(c k - b m)\ell _{1}^{2},\ \ \mathbf{g_{21}}=-e m\ell _{1}^{2},\ \ \mathbf{g_{22}}=f m\ell _{1}^{2}, \,\mathbf{g_{23}}= & {} -b m\ell _{1}^{2},\ \ \mathbf{g_{31}}=0,\ \ \mathbf{g_{32}}=-g m\ell _{1}^{2},\ \ \mathbf{g_{33}}=h m\ell _{1}^{2}. \end{aligned}$$

Here

$$\begin{aligned} \ell _{1}=\left[c e (g j k + h i l) + \left\{b (a - e) h i + \left(b (a - e) g - a f h\right) j\right\} m\right] \end{aligned}$$

Since $\det (\mathbb{D})>0$, and $\mathbf{g_{33}}>0$. Therefore $\mathbb{G}_{33}>0.$ $\square$

##### Lemma 4.7

The matrix denoted as *E*, which is defined in Lemma 4.4 as $\widetilde{\mathbb{C}}$, exhibits diagonal stability.

##### Proof

Since

$$\begin{aligned} \mathbf{c_{44}}=-(\mu (dgmn+bgmq) + c e g k q - a f h m q)<0, \end{aligned}$$

and

$$\begin{aligned} \det (-A)=-\mu m \left(d g j n + d h i p + b h i q + b g j q\right)-\left(c e (g j k + h i l) - a f h j m\right) q<0. \end{aligned}$$

So it is obvious that $E_{44}>0$, Using Lemma 4.2, our objective is to demonstrate that the reduced matrix $H=\widetilde{E}$ and $\mathbb{H}=\widetilde{\mathbb{E}^{-1}}$ exhibit diagonal stability. Thus the matrix *D* can be obtained from the matrix *E* given in Lemma 4.4, we have

$$\begin{aligned} H=\frac{1}{\det (-A)}\left( \begin{array}{ccc} \mathbf{{c}_{11}} &{} \mathbf{c_{12}} &{} \mathbf{c_{13}}\\ \mathbf{{c}_{21}} &{} \mathbf{c_{22}} &{} \mathbf{c_{23}}\\ \mathbf{{c}_{31}} &{} \mathbf{c_{32}} &{} \mathbf{c_{33}} \end{array}\right) ,\quad \textrm{and}\quad \,\mathbb{H}=\frac{1}{\det (E)}\left( \begin{array}{ccc} \mathbf{{h}_{11}} &{} \mathbf{h_{12}} &{} \mathbf{h_{13}}\\ \mathbf{{h}_{21}} &{} \mathbf{h_{22}} &{} \mathbf{h_{23}}\\ \mathbf{{h}_{31}} &{} \mathbf{h_{32}} &{} \mathbf{h_{33}} \end{array}\right) . \end{aligned}$$

Here

$$\begin{aligned} \det (E)=&\frac{-m q\ell ^{3}}{\det (-A)}>0, \end{aligned}$$

and

$$\begin{aligned} \mathbf{h_{11}}= & {} a m q\ell ^{2},\ \ \mathbf{h_{12}}=0,\ \ \mathbf{h_{13}}=(d m n - c k q + b m q)\ell ^{2},\ \ \mathbf{h_{21}}=-e m q\ell ^{2},\ \ \mathbf{h_{22}}=f m q\ell ^{2},\\ \mathbf{h_{23}}= & {} -m (d n + b q)\ell ^{2},\ \ \mathbf{h_{31}}=0,\ \ \mathbf{h_{32}}=-g m q\ell ^{2},\ \ \mathbf{h_{33}}=h m q\ell ^{2}. \end{aligned}$$

Since $\det (E)>0$, and $\mathbf{h_{33}}>0$. Therefore $\mathbb{H}_{33}>0.$ $\square$

##### Lemma 4.8

The matrix $\mathbb{E}$ given by $\widetilde{\mathbb{C}^{-1}}$ in Lemma 4.4 exhibits diagonal stability.

##### Proof

It is obvious that $\mathbb{E}_{44}>0$, by applying Lemma 4.2, our task is to establish the diagonal stability of the reduced matrix $I=\widetilde{\mathbb{E}}$ and its inverse $\mathbb{I}=\widetilde{\mathbb{E}^{-1}}$. This demonstration serves as the completion of the proof. Thus the matrix *I* and $\mathbb{I}$ can be obtained from the matrix $\mathbb{E}$ given in Lemma 4.4, we have

$$\begin{aligned} I=\frac{1}{\det (\mathbb{C})}\left( \begin{array}{ccc} \mathbf{{e}_{11}} &{} \mathbf{e_{12}} &{} \mathbf{e_{13}}\\ \mathbf{{e}_{21}} &{} \mathbf{e_{22}} &{} \mathbf{e_{23}}\\ \mathbf{{e}_{31}} &{} \mathbf{e_{32}} &{} \mathbf{e_{33}} \end{array}\right) ,\quad \textrm{and}\quad \,\mathbb{I}=\frac{1}{\det (\mathbb{E})}\left( \begin{array}{ccc} \mathbf{{i}_{11}} &{} \mathbf{i_{12}} &{} \mathbf{i_{13}}\\ \mathbf{{i}_{21}} &{} \mathbf{i_{22}} &{} \mathbf{i_{23}}\\ \mathbf{{i}_{31}} &{} \mathbf{i_{32}} &{} \mathbf{i_{33}} \end{array}\right) . \end{aligned}$$

Here

$$\begin{aligned} \det (\mathbb{E})=&\frac{(q\ell )^{3}}{\det (\mathbb{C})}\left[d (-a + e) (g j n + h i p) + \left\{b (-a + e) h i + \left((-a + e) b g + a f h\right) j\right\} q\right]>0, \end{aligned}$$

and

$$\begin{aligned} \mathbf{i_{11}}= & {} q^2 \left(d g j n + d h i p + b h i q + b g j q - f h j q\right)\ell ^{3},\\ \mathbf{i_{12}}= & {} q^2 \left(d g j n + d h i p + b h i q + b g j q\right)\ell ^{3},\\ \mathbf{i_{13}}= & {} f j q^2 \left(d n + b q\right)\ell ^{3},\ \ \mathbf{i_{21}}=e h j q^3\ell ^{3},\ \ \mathbf{i_{22}}=a h j q^3\ell ^{3},\\ \mathbf{i_{23}}= & {} -(a - e) j q^2 \left(d n + b q\right)\ell ^{3},\ \ \mathbf{i_{31}}=e g j q^3\ell ^{3},\ \ \mathbf{i_{32}}=a g j q^3\ell ^{3},\\ \mathbf{i_{33}}= & {} q^2 \left(d (a - e) i p + (b (a - e) i - a f j) q\right)\ell ^{3}. \end{aligned}$$

Since $\det (\mathbb{E})>0$, and $\mathbf{i_{33}}>0$. Therefore $\mathbb{I}_{33}>0.$ $\square$

##### Proposition 4.1

Let $a=\frac{\beta _{1}P}{1+\alpha _{1}P}+\frac{\beta _{2}(I_A+I_S)}{1+\alpha _{2}(I_A+I_S)}+\mu$, $b=\frac{\beta _{2}S^*}{(1+\alpha _{2}(I_A+I_S))(1+\alpha _{2}(I_A^*+I_S^*))}$, $f=(\mu +\upsilon )$, $g=\eta \upsilon$ and $h=(\mu +\delta +\gamma _S+\tau _{S})$ in (17), and if $(\mu +\delta +\gamma _S+\tau _{S})>\frac{\beta _{2}S^*}{(1+\alpha _{2}(I_A+I_S))(1+\alpha _{2}(I_A^*+I_S^*))}$, then the following conditions holds:

(i) $$fh>bg$$,
(ii) $$afh>\mu bg$$,
(iii) $$afhj>\mu b(hi+gj),$$
(iv) $$fhj>b(hi+gj),$$
(v) $$afj>\mu bi$$.

Assume that the pairs *fh*, *bg* and *afh* and $\mu bg$ are nearly equal, such that $bg-af\simeq 0$ and $\mu bg-afh\simeq 0$.

##### Proof

(*i*) Since

$$\begin{aligned} \upsilon =\upsilon ,\ \mu +\upsilon >\upsilon , \end{aligned}$$

as $0<\eta <1$, so $\mu +\upsilon >\eta \upsilon ,$ this implies that $f>g$. Furthermore, for

$$\begin{aligned} (\mu +\delta +\gamma _S+\tau _{S})>\frac{\beta _{2}S^*}{(1+\alpha _{2}(I_A+I_S))(1+\alpha _{2}(I_A^*+I_S^*))}, \end{aligned}$$

which implies that

$$\begin{aligned} h>b, \end{aligned}$$

therefore

$$\begin{aligned} (\mu +\upsilon )(\mu +\delta +\gamma _S+\tau _{S})>\frac{\beta _{2}S^*}{(1+\alpha _{2}(I_A+I_S))(1+\alpha _{2}(I_A^*+I_S^*))}\eta \upsilon . \end{aligned}$$

This implies that

$$\begin{aligned} fh>bg. \end{aligned}$$

(*ii*) One can easily see that

$$\begin{aligned} \frac{\beta _{1}P}{1+\alpha _{1}P}+\frac{\beta _{2}(I_A+I_S)}{1+\alpha _{2}(I_A+I_S)}+\mu >\mu , \end{aligned}$$

and for

$$\begin{aligned} (\mu +\delta +\gamma _S+\tau _{S})>\frac{\beta _{2}S^*}{(1+\alpha _{2}(I_A+I_S))(1+\alpha _{2}(I_A^*+I_S^*))}, \end{aligned}$$

from (*i*), we know that

$$\begin{aligned} (\mu +\upsilon )>\eta \upsilon . \end{aligned}$$

Therefore

$$\begin{aligned} \left(\frac{\beta _{1}P}{1+\alpha _{1}P}+\frac{\beta _{2}(I_A+I_S)}{1+\alpha _{2}(I_A+I_S)}+\mu \right)(\mu +\upsilon )(\mu +\delta +\gamma _S+\tau _{S})> \mu \frac{\beta _{2}S^*}{(1+\alpha _{2}(I_A+I_S))(1+\alpha _{2}(I_A^*+I_S^*))}\eta \upsilon , \end{aligned}$$

this implies that $afh>\mu bg.$

(*iii*) Since $a>\mu$ and $h>b$. This implies that

18 $$\begin{aligned} ah>\mu b. \end{aligned}$$

Now as $(\mu +\upsilon )>(1-\eta \upsilon )$ and $(\mu +\delta +\gamma _{A}+\tau _{A})>(\mu +\delta +\gamma _{S}+\tau _{S}).$ This implies that

$$\begin{aligned} (\mu +\upsilon )(\mu +\delta +\gamma _{A}+\tau _{A})>(1-\eta \upsilon )(\mu +\delta +\gamma _{S}+\tau _{S}), \end{aligned}$$

or

19 $$\begin{aligned} fj>hi. \end{aligned}$$

Also $(\mu +\upsilon )>\eta \upsilon$. Therefore $(\mu +\upsilon )(\mu +\delta +\gamma _{A}+\tau _{A})>\eta \upsilon (\mu +\delta +\gamma _{A}+\tau _{A})$. This implies that

20 $$\begin{aligned} fj>gj. \end{aligned}$$

From (19) and (20), we get

21 $$\begin{aligned} fj>(hi+gj). \end{aligned}$$

From (18) and (21), we deduced that

22 $$\begin{aligned} afhj>\mu b(hi+gj). \end{aligned}$$

(*iv*) From (i) and (iii), one can easily see that $fhj>b(hi+gj).$

(*v*) Since $a>\mu$, $(\mu +\upsilon )>(1-\eta )\upsilon$, and for

$$\begin{aligned} (\mu +\delta +\gamma _{A}+\tau _{A})>\frac{\beta _{2}S^*}{(1+\alpha _{2}(I_A+I_S))(1+\alpha _{2}(I_A^*+I_S^*))}. \end{aligned}$$

This implies that

$$\begin{aligned} a(\mu +\upsilon )(\mu +\delta +\gamma _{A}+\tau _{A})>\mu (1-\eta )\upsilon \frac{\beta _{2}S^*}{(1+\alpha _{2}(I_A+I_S))(1+\alpha _{2}(I_A^*+I_S^*))}, \end{aligned}$$

which means that

23 $$\begin{aligned} afh>\mu bi. \end{aligned}$$

$$\square$$

##### Lemma 4.9

The matrix denoted as *F* and defined as $\widetilde{D}$ in Lemma 4.5 exhibits diagonal stability.

##### Proof

It is obvious that $F_{33}>0$, using Lemma 4.1, we must establish the diagonal stability of both the reduced matrix $J=\widetilde{F}$ and the matrix $\mathbb{J}=\widetilde{F^{-1}}$. Thus from Lemma 4.5 we have

$$\begin{aligned} J=\left( \begin{array}{cc} a &{} 0\\ -e &{} f\\ \end{array}\right) . \end{aligned}$$

Since both $J_{11}>0$ and $J_{22}>0$, furthermore $\det (J)=af>0$ because both *a* and *f* are positive. Now

$$\begin{aligned} F^{-1}= & {} \frac{1}{-\mu bg+afh}\left( \begin{array}{ccc} -bg+fh &{} -bg &{} -bf\\ eh &{} ah &{} b(a-e)\\ eg &{} ag &{} af \end{array}\right) ,\\ \mathbb{J}= & {} \frac{1}{-\mu bg+afh}\left( \begin{array}{cc} -bg+fh &{} -bg\\ eh &{} ah \end{array}\right) . \end{aligned}$$

Since

$$\begin{aligned} \mathbb{J}_{11}=\frac{-bg+fh}{-\mu bg+afh}>0,\,\, \mathbb{J}_{22}=\frac{ah}{-\mu bg+afh}>0,\,\quad \textrm{and}\quad \,\det (\mathbb{J})=\frac{h}{-\mu bg+afh}>0\,\, \textrm{by}\, \textrm{Proposition}\,4.1. \end{aligned}$$

Thus using Lemma 4.1, the matrix *F* is diagonally stable. $\square$

##### Lemma 4.10

The matrix $\mathbb{F}$, as defined in Lemma 4.5 with $\widetilde{D^{-1}}$, exhibits diagonal stability.

##### Proof

It is obvious that $\mathbb{F}_{33}>0$, applying Lemma 4.1, our goal is to establish the diagonal stability for both the reduced matrix $K=\widetilde{\mathbb{F}}$ and the matrix $\mathbb{K}=\widetilde{\mathbb{F}^{-1}}$. Thus from Lemma 4.5 we have

$$\begin{aligned} K=\frac{1}{\det ({D})}\left( \begin{array}{cc} \mathbf{f_{11}} &{} \mathbf{f_{12}}\\ \mathbf{f_{21}} &{} \mathbf{f_{22}} \end{array}\right) . \end{aligned}$$

Since

$$\begin{aligned} K_{11}=\frac{\mathbf{f_{11}}}{\det (D)}>0 \,\,\quad \textrm{and}\quad \,\, K_{22}=\frac{\mathbf{f_{22}}}{\det (D)}>0. \end{aligned}$$

Furthermore

$$\begin{aligned} K_{21}=\frac{\mathbf{f_{21}}}{\det (D)}>0\,\, \quad \textrm{and}\quad \,\, K_{12}=\frac{\mathbf{f_{12}}}{\det (D)}<0 \,\,\textrm{see} \,\,4.5,\,\, \textrm{therefore}\,\,\det (K)>0. \end{aligned}$$

Now

$$\begin{aligned} \mathbb{K}=\frac{1}{\det (\mathbb{F})}\left( \begin{array}{cc} \mathbf{k_{11}} &{} \mathbf{k_{12}}\\ \mathbf{k_{21}} &{} \mathbf{k_{22}} \end{array}\right) , \end{aligned}$$

here

$$\begin{aligned} \det (\mathbb{F})=\frac{j \left\{b (-a + e) h i + \left((-a + e)b g + a f h\right) j\right\}^2}{\det (D)}>0, \end{aligned}$$

and

$$\begin{aligned} \mathbf{k_{11}}= & {} a j \left(b (-a + e) h i + ((-a + e)b g + a f h) j\right)>0\ \ (\textrm{see}\ \ \textrm{Proposition} \ \ 4.1\ \ (iii)),\\ \mathbf{k_{12}}= & {} a b f g j^2 + b \left(b (-a + e) i + a f j\right) (h i + g j)>0 \ \ (\textrm{see}\ \ \textrm{Proposition} \ \ 4.1\ \ (v)),\\ \mathbf{k_{21}}= & {} -e j \left(b (-a + e) h i + ((-a + e)b g + a f h) j\right)<0 \ \ (\textrm{see}\ \ \textrm{Proposition} \ \ 4.1\ \ (iii)),\ \\ \mathbf{k_{22}}= & {} -b e f g j^2 + \left(b (-a + e) i + a f j\right) \left(f h j - b (h i + g j)\right)>0. \ \ (\textrm{see}\ \ \textrm{Proposition} \ \ 4.1\ \ (iv) \ \ \quad \textrm{and}\quad \ \ (v)). \end{aligned}$$

Since

$$\begin{aligned} \mathbb{K}_{11}=\frac{\mathbf{k_{11}}}{\det (\mathbb{F})}>0 \,\,\quad \textrm{and}\quad \,\, \mathbb{K}_{22}=\frac{\mathbf{k_{22}}}{\det (\mathbb{F})}>0, \end{aligned}$$

furthermore

$$\begin{aligned} \mathbb{K}_{21}=\frac{\mathbf{k_{21}}}{\det (\mathbb{F})}<0\,\, \quad \textrm{and}\quad \,\, \mathbb{K}_{12}=\frac{\mathbf{k_{12}}}{\det (\mathbb{F})}>0. \end{aligned}$$

Therefore

$$\begin{aligned} \det (\mathbb{K})=\frac{\mathbf{k_{11}}\mathbf{k_{22}}-\mathbf{k_{21}}\mathbf{k_{12}}}{\det (\mathbb{F})}>0. \end{aligned}$$

Thus using Lemma 4.1, the matrix $\mathbb{F}$ is diagonally stable. $\square$

##### Lemma 4.11

The matrix *G* given by $\widetilde{\mathbb{D}}$ in Lemma 4.6 exhibits diagonal stability.

##### Proof

It is obvious that $G_{33}>0$, applying Lemma 4.1, our goal is to establish the diagonal stability of both the reduced matrix $L=\widetilde{G}$ and $\mathbb{L}=\widetilde{G^{-1}}$. Thus from Theorem 4.6 we have

$$\begin{aligned} L=\frac{1}{\det (C)}\left( \begin{array}{cc} \mathbf{d_{11}} &{} \mathbf{d_{12}}\\ \mathbf{d_{21}} &{} \mathbf{d_{22}}\\ \end{array}\right) . \end{aligned}$$

Since

$$\begin{aligned} L_{11}=\frac{\mathbf{d_{11}}}{\det (C)}>0 \,\,\quad \textrm{and}\quad \,\, L_{22}=\frac{\mathbf{d_{22}}}{\det (C)}>0, \end{aligned}$$

furthermore

$$\begin{aligned} L_{21}=\frac{\mathbf{d_{21}}}{\det (C)}>0\,\, \quad \textrm{and}\quad \,\, L_{12}=\frac{\mathbf{d_{12}}}{\det (C)}<0 \,\,(\textrm{see}\,\, \textrm{Lemma} \,\,4.3\,\,\textrm{for} \,\,d_{ij}\,\, \quad \textrm{and}\quad \,\, \det (C)). \end{aligned}$$

Therefore

$$\begin{aligned} \det (L)=\frac{\mathbf{d_{11}}\mathbf{d_{22}}-\mathbf{d_{21}}\mathbf{d_{12}}}{\det (C)}>0. \end{aligned}$$

Now

$$\begin{aligned} \mathbb{L}=\frac{1}{\det (G)}\left( \begin{array}{cc} \mathbf{l_{11}} &{} \mathbf{l_{12}}\\ \mathbf{l_{21}} &{} \mathbf{l_{22}} \end{array}\right) , \end{aligned}$$

here

$$\begin{aligned} \det (G)=\frac{j m \ell _{1}^2}{\det (C)}<0, \end{aligned}$$

and

$$\begin{aligned} \mathbf{l_{11}}= & {} -a j m\ell _{1}<0,\ \ \mathbf{l_{12}}=i (c l - b m)\ell _{1}<0 \,\,\textrm{if}\,\, c l< b m,\\ \mathbf{l_{21}}= & {} e j m\ell _{1}>0, \ \ \mathbf{l_{22}}=(b i - f j) m\ell _{1}<0 \,\,\textrm{if}\,\, b i<f j. \end{aligned}$$

Since

$$\begin{aligned} \mathbb{L}_{11}=\frac{\mathbf{l_{11}}}{\det (G)}>0 \,\,\quad \textrm{and}\quad \,\, \mathbb{L}_{22}=\frac{\mathbf{l_{22}}}{\det (G)}>0, \end{aligned}$$

furthermore

$$\begin{aligned} \mathbb{L}_{21}=\frac{\mathbf{l_{21}}}{\det (G)}>0\,\, \quad \textrm{and}\quad \,\, \mathbb{L}_{12}=\frac{\mathbf{l_{12}}}{\det (G)}<0. \end{aligned}$$

Therefore

$$\begin{aligned} \det (\mathbb{L})=\frac{\mathbf{l_{11}}\mathbf{l_{22}}-\mathbf{l_{21}}\mathbf{l_{12}}}{\det (G)}>0. \end{aligned}$$

Thus using Lemma 4.1, the matrix *G* is diagonally stable. $\square$

##### Lemma 4.12

The matrix $\mathbb{G}$ is given by $\widetilde{\mathbb{D}^{-1}}$ in Lemma 4.6 exhibits diagonal stability.

##### Proof

It is obvious that $\mathbb{G}_{33}>0$, applying Lemma 4.1, our goal is establish the diagonal stability of both the reduced matrix $M=\widetilde{\mathbb{G}}$ and $\mathbb{M}=\widetilde{\mathbb{G}^{-1}}$. Thus from Lemma 4.6 we have

$$\begin{aligned} M=\frac{1}{\det ({\mathbb{D}})}\left( \begin{array}{cc} \mathbf{g_{11}} &{} \mathbf{g_{12}}\\ \mathbf{g_{21}} &{} \mathbf{g_{22}} \end{array}\right) . \end{aligned}$$

Since

$$\begin{aligned} M_{11}=\frac{\mathbf{g_{11}}}{\det (\mathbb{D})}>0 \,\,\quad \textrm{and}\quad \,\, M_{22}=\frac{\mathbf{g_{22}}}{\det (\mathbb{D})}>0, \end{aligned}$$

furthermore

$$\begin{aligned} M_{21}=\frac{\mathbf{g_{21}}}{\det (\mathbb{D})}>0\,\, \quad \textrm{and}\quad \,\, M_{12}=\frac{\mathbf{g_{12}}}{\det (\mathbb{D})}<0 \,\,(\textrm{see}\,\, \textrm{Lemma} \,\,4.6\,\,\textrm{for} \,\,g_{ij}\,\, \quad \textrm{and}\quad \,\, \det ({\mathbb{D}})). \end{aligned}$$

Therefore

$$\begin{aligned} \,\,\det (M)=\frac{\mathbf{g_{11}}\mathbf{g_{22}} -\mathbf{g_{21}}\mathbf{g_{12}}}{\det ({\mathbb{D}})}>0. \end{aligned}$$

Now

$$\begin{aligned} \mathbb{M}=\frac{1}{\det (\mathbb{G})}\left( \begin{array}{cc} \mathbf{m_{11}} &{} \mathbf{m_{12}}\\ \mathbf{m_{21}} &{} \mathbf{m_{22}} \end{array}\right) , \end{aligned}$$

here

$$\begin{aligned} \det (\mathbb{G})=\frac{m^2 \left(-c e g k + \left((-a + e)b g + a f h\right) m\right)\ell _{1}^6}{\det (\mathbb{D})}>0, \end{aligned}$$

and

$$\begin{aligned} \mathbf{m_{11}}= & {} (-b g + f h) m^2\ell _{1}^4>0,\ \ \mathbf{m_{12}}=-g m (-c k + b m)\ell _{1}^4<0,\\ \mathbf{m_{21}}= & {} e h m^2\ell _{1}^4>0,\ \ \mathbf{m_{22}}=a h m^2\ell _{1}^4>0. \end{aligned}$$

Since

$$\begin{aligned} \mathbb{M}_{11}=\frac{\mathbf{m_{11}}}{\det (\mathbb{G})}>0 \,\, \quad \textrm{and}\quad \,\, \mathbb{M}_{22}=\frac{\mathbf{m_{22}}}{\det (\mathbb{G})}>0, \end{aligned}$$

furthermore

$$\begin{aligned} \mathbb{G}_{21}=\frac{\mathbf{m_{21}}}{\det (\mathbb{G})}>0\,\, \quad \textrm{and}\quad \,\, \mathbb{M}_{12}=\frac{\mathbf{m_{12}}}{\det (\mathbb{G})}<0. \end{aligned}$$

Therefore

$$\begin{aligned}\det (\mathbb{M})=\frac{\mathbf{m_{11}}\mathbf{m_{22}} -\mathbf{m_{21}}\mathbf{m_{12}}}{\det (\mathbb{G})}>0. \end{aligned}$$

Thus using Lemma 4.1, the matrix $\mathbb{M}$ is diagonally stable. $\square$

##### Lemma 4.13

The matrix *H* given by $\widetilde{E}$ in Lemma 4.7 exhibits diagonal stability.

##### Proof

It is obvious that $H_{33}>0$, applying Lemma 4.1, our goal is to establish the diagonal stability of both the reduced matrix $N=\widetilde{H}$ and the matrix $\mathbb{N}=\widetilde{H^{-1}}$. Thus from Lemma 4.7 we have

$$\begin{aligned} N=\frac{1}{\det (-A)}\left( \begin{array}{cc} \mathbf{c_{11}} &{} \mathbf{c_{12}}\\ \mathbf{c_{21}} &{} \mathbf{c_{22}} \end{array}\right) . \end{aligned}$$

Since

$$\begin{aligned} N_{11}=\frac{\mathbf{c_{11}}}{\det (-A)}>0 \,\,\quad \textrm{and}\quad \,\, N_{22}=\frac{\mathbf{c_{22}}}{\det (-A)}>0, \end{aligned}$$

furthermore

$$\begin{aligned}N_{21}=\frac{\mathbf{c_{21}}}{\det (-A)}<0\,\, \quad \textrm{and}\quad \,\, N_{12}=\frac{\mathbf{c_{12}}}{\det (-A)}>0 \,\,(\textrm{see}\,\, \textrm{Theorem} \,\,4.6\,\,\textrm{for} \,\,c_{ij}\,\, \quad \textrm{and}\quad \,\, \det (-A)). \end{aligned}$$

Therefore

$$\begin{aligned} \,\,\det (N)=\frac{\mathbf{c_{11}}\mathbf{c_{22}}-\mathbf{c_{21}}\mathbf{c_{12}}}{\det (-A)}>0. \end{aligned}$$

Now

$$\begin{aligned} \mathbb{N}=\frac{1}{\det (H)}\left( \begin{array}{cc} \mathbf{n_{11}} &{} \mathbf{n_{12}}\\ \mathbf{n_{21}} &{} \mathbf{n_{22}} \end{array}\right) , \end{aligned}$$

here

$$\begin{aligned} \det (H)=\frac{j m q\ell ^2}{\det (-A)}<0, \end{aligned}$$

and $$\mathbf{n_{11}}=-a j m q\ell <0,$$$$\mathbf{n_{12}}=-i (d m p - c l q + b m q)\ell <0,$$$$\mathbf{n_{21}}=e j m q\ell >0,$$$$\mathbf{n_{22}}=m (d i p + b i q - f j q)\ell <0 \,\,\textrm{if} \,\,f j q>d i p + b i q.$$

Since

$$\begin{aligned} \mathbb{N}_{11}=\frac{\mathbf{n_{11}}}{\det (H)}>0 \,\,\quad \textrm{and}\quad \,\, \mathbb{N}_{22}=\frac{\mathbf{n_{22}}}{\det (H)}>0, \end{aligned}$$

furthermore

$$\begin{aligned} \mathbb{N}_{21}=\frac{\mathbf{n_{21}}}{\det (H)}<0\,\, \quad \textrm{and}\quad \,\, \mathbb{N}_{12}=\frac{\mathbf{n_{12}}}{\det (H)}>0. \end{aligned}$$

Therefore

$$\begin{aligned} \det (\mathbb{N})=\frac{\mathbf{n_{11}}\mathbf{n_{22}}-\mathbf{n_{21}}\mathbf{n_{12}}}{\det (H)}>0. \end{aligned}$$

Thus using Lemma 4.1, the matrix *H* is diagonally stable. $\square$

##### Lemma 4.14

The matrix $\mathbb{H}$ given by $\widetilde{E^{-1}}$ in Lemma 4.7 exhibits diagonal stability.

##### Proof

It is obvious that $\mathbb{H}_{33}>0$, applying Lemma 4.1, our goal is to establish the diagonal stability of both the reduced matrix $P=\widetilde{\mathbb{H}}$ and $\mathbb{P}=\widetilde{\mathbb{H}^{-1}}$. Thus from Lemma 4.7, one has

$$\begin{aligned} P=\frac{1}{\det (E)}\left( \begin{array}{cc} \mathbf{h_{11}} &{} \mathbf{h_{12}}\\ \mathbf{h_{21}} &{} \mathbf{h_{22}} \end{array}\right) . \end{aligned}$$

Since

$$\begin{aligned} P_{11}=\frac{\mathbf{h_{11}}}{\det (E)}>0 \,\,\quad \textrm{and}\quad \,\, P_{22}=\frac{\mathbf{h_{22}}}{\det (E)}>0, \end{aligned}$$

furthermore

$$\begin{aligned} P_{21}=\frac{\mathbf{h_{21}}}{\det (E)}<0\,\, \quad \textrm{and}\quad \,\, P_{12}=\frac{\mathbf{h_{12}}}{\det (E)}=0 \,\,(\textrm{see}\,\, \textrm{Lemma} \,\,4.7\,\,\textrm{for} \,\,h_{ij} \,\, \quad \textrm{and}\quad \,\, \det (E)). \end{aligned}$$

Therefore

$$\begin{aligned} \det (P)=\frac{\mathbf{h_{11}}\mathbf{h_{22}}-\mathbf{h_{21}}\mathbf{h_{12}}}{\det (E)}>0. \end{aligned}$$

Now

$$\begin{aligned} \mathbb{P}=\frac{1}{\det (\mathbb{H})}\left( \begin{array}{cc} \mathbf{p_{11}} &{} \mathbf{p_{12}}\\ \mathbf{p_{21}} &{} \mathbf{p_{22}} \end{array}\right) , \end{aligned}$$

here

$$\begin{aligned} \det (\mathbb{H})=\frac{m^2 q^2 \left(d (a - e) g m n + (c e g k + (b (a - e) g - a f h) m) q\right)\ell ^6}{\det (E)}>0, \end{aligned}$$

where

$$\begin{aligned} \mathbf{p_{11}}= & {} m^2 q\ell ^4 (f h q-g (d n + b q))>0,\ \ \mathbf{p_{12}}=-g m q (d m n - c k q + b m q)\ell ^4<0,\\ \mathbf{p_{21}}= & {} e h m^2 q^2\ell ^4>0,\ \ \mathbf{p_{22}}=a h m^2 q^2\ell ^4>0. \end{aligned}$$

Since

$$\begin{aligned} \mathbb{P}_{11}=\frac{\mathbf{p_{11}}}{\det (\mathbb{H})}>0 \,\,\quad \textrm{and}\quad \,\, \mathbb{P}_{22}=\frac{\mathbf{p_{22}}}{\det (\mathbb{H})}>0, \end{aligned}$$

furthermore

$$\begin{aligned} \mathbb{P}_{21}=\frac{\mathbf{p_{21}}}{\det (\mathbb{H})}>0\,\, \quad \textrm{and}\quad \,\, \mathbb{P}_{12}=\frac{\mathbf{p_{12}}}{\det (\mathbb{H})}<0. \end{aligned}$$

Therefore

$$\begin{aligned} \det (\mathbb{P})=\frac{\mathbf{p_{11}}\mathbf{p_{22}}-\mathbf{p_{21}}\mathbf{p_{12}}}{\det (\mathbb{H})}>0. \end{aligned}$$

Thus using Lemma 4.1, the matrix $\mathbb{H}$ is diagonally stable. $\square$

##### Lemma 4.15

The matrix *I* given by $\widetilde{\mathbb{E}}$ in Lemma 4.9 exhibits diagonal stability.

##### Proof

It is obvious that $I_{33}>0$, applying Lemma 4.1, our goal is to establish diagonal stability of both the reduced matrix $Q=\widetilde{I}$ and $\mathbb{Q}=\widetilde{I^{-1}}$. Thus from Lemma 4.9 we have

$$\begin{aligned} Q=\frac{1}{\det ({\mathbb{C}})}\left( \begin{array}{cc} \mathbf{e_{11}} &{} \mathbf{e_{12}}\\ \mathbf{e_{21}} &{} \mathbf{e_{22}} \end{array}\right) . \end{aligned}$$

Since

$$\begin{aligned} Q_{11}=\frac{\mathbf{e_{11}}}{\det (\mathbb{C})}>0 \,\,\quad \textrm{and}\quad \,\, Q_{22}=\frac{\mathbf{e_{22}}}{\det (\mathbb{C})}>0, \end{aligned}$$

furthermore

$$\begin{aligned} Q_{21}=\frac{\mathbf{e_{21}}}{\det (\mathbb{C})}>0\,\, \quad \textrm{and}\quad \,\, Q_{12}=\frac{\mathbf{e_{12}}}{\det (\mathbb{C})}=0 \,\,(\textrm{see}\,\, \textrm{Lemma} \,\,4.4\,\,\textrm{for} \,\,e_{ij}\,\, \quad \textrm{and}\quad \,\, \det ({\mathbb{C}})). \end{aligned}$$

Therefore,

$$\begin{aligned} \det (Q)=\frac{\mathbf{e_{11}}\mathbf{e_{22}}-\mathbf{e_{21}}\mathbf{e_{12}}}{\det ({\mathbb{C}})}>0. \end{aligned}$$

Now

$$\begin{aligned} \mathbb{Q}=\frac{1}{\det (I)}\left( \begin{array}{cc} \mathbf{q_{11}} &{} \mathbf{q_{12}}\\ \mathbf{q_{21}} &{} \mathbf{q_{22}} \end{array}\right) , \end{aligned}$$

here

$$\begin{aligned} \det (I)=\frac{q^2 \left(d (a - e) g n + (b (a - e) g - a f h) q\right)\ell ^3}{\det (\mathbb{C})}, \end{aligned}$$

where

$$\begin{aligned} \mathbf{q_{11}}= & {} -q (d g n + b g q - f h q) \ell ^2<0,\ \ \mathbf{q_{12}}=-g q (d n + b q) \ell ^2<0,\\ \mathbf{q_{21}}= & {} e h q^2 \ell ^2>0,\ \ \mathbf{q_{22}}=-a h q^2 \ell ^2<0. \end{aligned}$$

Since

$$\begin{aligned} \mathbb{Q}_{11}=\frac{\mathbf{q_{11}}}{\det (I)}>0 \,\,\quad \textrm{and}\quad \,\, \mathbb{Q}_{22}=\frac{\mathbf{q_{22}}}{\det (I)}>0, \end{aligned}$$

furthermore

$$\begin{aligned} \mathbb{Q}_{21}=\frac{\mathbf{q_{21}}}{\det (I)}>0\,\, \quad \textrm{and}\quad \,\, \mathbb{Q}_{12}=\frac{\mathbf{q_{12}}}{\det (I)}<0. \end{aligned}$$

Therefore

$$\begin{aligned} \det (\mathbb{Q})=\frac{\mathbf{q_{11}}\mathbf{q_{22}}-\mathbf{q_{21}}\mathbf{q_{12}}}{\det (I)}>0. \end{aligned}$$

Thus using Lemma 4.1, the matrix *I* is diagonally stable. $\square$

##### Lemma 4.16

The matrix $\mathbb{I}$ given by $\widetilde{\mathbb{E}^{-1}}$ in Lemma 4.8 exhibits diagonal stability.

##### Proof

It is obvious that $\mathbb{I}_{33}>0$, applying Lemma 4.1, our goal is to establish diagonal stability of both the reduced matrix $T=\widetilde{\mathbb{I}}$ and the matrix $\mathbb{T}=\widetilde{\mathbb{I}^{-1}}$. Thus from Lemma 4.8 we have

$$\begin{aligned} T=\frac{1}{\det ({\mathbb{E}})}\left( \begin{array}{cc} \mathbf{i_{11}} &{} \mathbf{i_{12}}\\ \mathbf{i_{21}} &{} \mathbf{i_{22}} \end{array}\right) . \end{aligned}$$

Since

$$\begin{aligned} T_{11}=\frac{\mathbf{i_{11}}}{\det (\mathbb{E})}>0 \,\,\quad \textrm{and}\quad \,\, T_{22}=\frac{\mathbf{i_{22}}}{\det (\mathbb{E})}>0, \end{aligned}$$

furthermore

$$\begin{aligned} T_{21}=\frac{\mathbf{i_{21}}}{\det (\mathbb{E})}<0\,\, \quad \textrm{and}\quad \,\, T_{12}=\frac{\mathbf{i_{12}}}{\det (\mathbb{E})}>0 \,\,(\textrm{see}\,\, \textrm{Lemma} \,\,4.8\,\,\textrm{for} \,\,i_{ij}\,\, \quad \textrm{and}\quad \,\, \det ({\mathbb{E}}). \end{aligned}$$

Therefore

$$\begin{aligned} \,\,\det (T)=\frac{\mathbf{i_{11}}\mathbf{i_{22}}-\mathbf{i_{21}} \mathbf{i_{12}}}{\det ({\mathbb{E}})}>0. \end{aligned}$$

Now

$$\begin{aligned} \mathbb{T}=\frac{1}{\det (\mathbb{I})}\left( \begin{array}{cc} \mathbf{t_{11}} &{} \mathbf{t_{12}}\\ \mathbf{t_{21}} &{} \mathbf{t_{22}} \end{array}\right) , \end{aligned}$$

here

$$\begin{aligned} \det (\mathbb{I})=\frac{j q^7 \left(d (-a + e) (g j n + h i p) + (b (-a + e) h i + (-a b g + b e g + a f h) j) q\right)^2 \ell ^3}{\det (\mathbb{E})}>0, \end{aligned}$$

and

$$\begin{aligned} \textbf{t}_{11}&=a j q^5 \left((a - e) g j (d n + b q) + h (d (a - e) i p + (b (a - e) i - a f j) q)\right) \ell ^2>0,\\ \textbf{t}_{12}&=q^4 \left(a f g j^2 q (d n + b q) - (d g j n + d h i p + b h i q + b g j q) (d (a - e) i p + (b (a - e) i - a f j) q)\right) \ell ^2>0,\\ \textbf{t}_{21}&=e j q^5 \left((-a + e) g j (d n + b q) + h (d (-a + e) i p + ((-a + e)b i + a f j) q)\right) \ell ^2<0,\\ \textbf{t}_{22}&=q^4 \left(-e f g j^2 q (d n + b q) + (d g j n + d h i p + b h i q + b g j q - f h j q) (d (a - e) i p + (b (a - e) i - a f j) q)\right) \ell ^2>0. \end{aligned}$$

Since

$$\begin{aligned} \mathbb{T}_{11}=\frac{\mathbf{t_{11}}}{\det (\mathbb{I})}>0 \,\,\quad \textrm{and}\quad \,\, \mathbb{T}_{22}=\frac{\mathbf{t_{22}}}{\det (\mathbb{I})}>0, \end{aligned}$$

furthermore

$$\begin{aligned} \mathbb{T}_{21}=\frac{\mathbf{m_{21}}}{\det (\mathbb{I})}<0\,\, \quad \textrm{and}\quad \,\, \mathbb{T}_{12}=\frac{\mathbf{t_{12}}}{\det (\mathbb{I})}>0. \end{aligned}$$

Therefore

$$\begin{aligned} \det (\mathbb{T})=\frac{\mathbf{t_{11}}\mathbf{t_{22}} -\mathbf{t_{21}}\mathbf{t_{12}}}{\det (\mathbb{I})}>0. \end{aligned}$$

Thus using Lemma 4.1, the matrix $\mathbb{I}$ is diagonally stable. $\square$

In summary of the preceding discussions, we’ve drawn these conclusions regarding the global asymptotic stability of the EE.

##### Theorem 4.7

When the value of $R_{0}$ is greater than 1, the endemic equilibrium $E_{*}=(S^*, E^*, I_{S}^*, I_{A}^*, R^*, P^*)$ in model (2) exhibits global asymptotic stability within $\Omega$.

##### Proof

Building upon above Theorem and a sequence of lemmas, it is assured that the EE of model (2) achieves global asymptotic stability. $\square$

## Sensitivity analysis

Here in this section, we conduct sensitivity analysis to explore how the model responds to variations in parameter values, aiming to understand which parameters significantly influence the transmission of the disease, specifically the reproductive number ($R_{0}$). To perform this analysis, we employ the normalized forward sensitivity index method, as outlined in^52^. This method involves determining the ratio of the relative change in a variable to the relative change in the corresponding parameter. Alternatively, sensitivity indices can be derived using partial derivatives when the variable is a differentiable function of the parameter.

Therefore, for our proposed model, we present the normalized forward sensitivity index of “$R_{0}$” using the given role:

$$\begin{aligned} S^{R_{0}}_{n}=\left[\frac{\partial R_{0}}{\partial n}\right]\times \frac{n}{R_{0}}. \end{aligned}$$

24 $$\begin{aligned} S^{R_{0}}_{b}&=\frac{1}{2R_{0}}\left\{R_{0}^{H}+\frac{(R_{0}^{H})^{2}+2R_{0}^{P}}{\sqrt{(R_{0}^{H})^{2}+4R_{0}^{P}}}\right\}>0,\\ S^{R_{0}}_{\mu }&=-\frac{\mu }{R_{0}}\left[\frac{b\upsilon }{2(\mu c_{1})^{2}}\left\{\beta _{2}\left(\frac{\kappa _{1}}{c_{2}^{2}}+\frac{\kappa _{2}}{c_{3}^{2}}\right)+\frac{R_{0}^{H}\beta _{2}\mu _{P}\left(\frac{\kappa _{1}}{c_{2}^{2}}+\frac{\kappa _{2}}{c_{3}^{2}}\right)+2\beta _{1}\left(\frac{\tau _{S}\kappa _{1}}{c_{2}^{2}}+ \frac{\tau _{S}\kappa _{2}}{c_{3}^{2}}\right)}{\mu _{P}\sqrt{(R_{0}^{H})^{2}+4R_{0}^{P}}}\right\}\right]<0,\\ \textrm{here}\,\, \kappa _{1}&=\eta (\mu c_{1}+c_{2}(c_{1}+\mu )) \,\,\quad \textrm{and}\quad \,\,\kappa _{2}=(1-\eta )(\mu c_{1}+c_{3}(c_{1}+\mu ))\\ S^{R_{0}}_{\mu _{P}}&=-\frac{1}{R_{0}}\left\{\frac{R_{0}^{P}}{\sqrt{(R_{0}^{H})^{2}+4R_{0}^{P}}}\right\}<0,\\ S^{R_{0}}_{\upsilon }&=\frac{1}{2R_{0}}\left\{R_{0}^{H}+\frac{(R_{0}^{H})^{2}+4R_{0}^{P}}{\sqrt{(R_{0}^{H})^{2}+4R_{0}^{P}}}\right\}>0,\\ S^{R_{0}}_{\eta }&=\frac{\eta }{2R_{0}}\left\{\frac{b\upsilon }{\mu c_{1}}\left(\beta _{2}(\frac{1}{c_{2}}-\frac{1}{c_{3}})+ \frac{R_{0}^{H}\beta _{2}(\frac{1}{c_{2}}-\frac{1}{c_{3}})+\frac{2\beta _{1}}{\mu _{P}}(\frac{\tau _{S}}{c_{2}}-\frac{\tau _{A}}{c_{3}})}{\sqrt{(R_{0}^{H})^{2}+4R_{0}^{P}}}\right)\right\}<0 \,\,\textrm{if}\,\, c_{2}>c_{3},\\ S^{R_{0}}_{\delta }&=-\frac{\delta }{2R_{0}}\left[\frac{b\upsilon }{\mu c_{1}}\left\{\beta _{2}\left(\frac{\eta }{c_{2}^{2}}+\frac{1-\eta }{c_{3}^{2}}\right)+\frac{R_{0}^{H}\beta _{2}\mu _{P}\left(\frac{\eta }{c_{2}^{2}}+\frac{1-\eta }{c_{3}^{2}}\right)+ 2\beta _{1}\left(\frac{\tau _{S}\eta }{c_{2}^{2}}+\frac{\tau _{A}(1-\eta )}{c_{3}^{2}}\right)}{\mu _{P}\sqrt{(R_{0}^{H})^{2}+4R_{0}^{P}}}\right\}\right]<0,\\ S^{R_{0}}_{\beta _{1}}&=\frac{1}{R_{0}}\left\{\frac{R_{0}^{P}}{\sqrt{(R_{0}^{H})^{2}+4R_{0}^{P}}}\right\}>0,\\ S^{R_{0}}_{\beta _{2}}&=\frac{1}{2R_{0}}\left\{R_{0}^{H}+\frac{(R_{0}^{H})^{2}}{\sqrt{(R_{0}^{H})^{2}+4R_{0}^{P}}}\right\}>0,\\ S^{R_{0}}_{\gamma _{S}}&=-\frac{\gamma _{S}}{2R_{0}}\left\{\frac{b\upsilon \eta }{\mu c_{1}c_{2}^{2}}\left(\beta _{2}+\frac{R_{0}^{H}\mu _{P}\beta _{2}+2\beta _{1}\tau _{S}}{\mu _{P}\sqrt{(R_{0}^{H})^{2}+4R_{0}^{P}}}\right)\right\}<0,\\ S^{R_{0}}_{\gamma _{A}}&=-\frac{\gamma _{A}}{2R_{0}}\left\{\frac{b\upsilon (1-\eta )}{\mu c_{1}c_{3}^{2}}\left(\beta _{2}+\frac{R_{0}^{H}\mu _{P}\beta _{2}+2\beta _{1}\tau _{A}}{\mu _{P}\sqrt{(R_{0}^{H})^{2}+4R_{0}^{P}}}\right)\right\}<0,\\ S^{R_{0}}_{\tau _{S}}&=-\frac{\tau _{S}}{2R_{0}}\left\{\frac{b\eta \upsilon }{\mu c_{1}c_{2}^{2}}\left(\beta _{2}+\frac{R_{0}^{H}\mu _{P}\beta _{2}+2\beta _{1}\tau _{S}}{\mu _{P}\sqrt{(R_{0}^{H})^{2}+4R_{0}^{P}}}\right)\right\}<0,\\ S^{R_{0}}_{\tau _{A}}&=-\frac{\tau _{A}}{2R_{0}}\left\{\frac{b\upsilon (1-\eta )}{\mu c_{1}c_{3}^{2}}\left(\beta _{2}+\frac{R_{0}^{H}\mu _{P}\beta _{2}+2\beta _{1}\tau _{A}}{\mu _{P}\sqrt{(R_{0}^{H})^{2}+4R_{0}^{P}}}\right)\right\}<0. \end{aligned}$$

## Numerical results and discussion

We simulate our results by extending the NSFD method. The mentioned scheme is convergent, consisted and have many dynamical properties (see^32^). Mickens’s^34^ established a general NSFD method for a coupled system. The reason behind of develing such schemes was that to avoid inaccuracies in the standard methods like Euler, RKM methods. Here, consider a problem^35^ described by

25 $$\begin{aligned} Dy=\frac{dy}{dt}=\theta (t, y(t)),\ y(0)=y_0, \end{aligned}$$

we define $t_{i+1}=t_0+nh$, where *h* is positive step size, let the discretization of *y* at $t_n$ be $y_n\approx y(t_n)$, then the the problem (25) becomes

26 $$\begin{aligned} D_hy(t_n)=\Phi _n(t_n, \theta , y_n). \end{aligned}$$

The procedure (26) is called NSFD method, because

- In the discretized version of (26), the usual denominator *h* is replaced by a nonnegative function say $\phi (h);$ such that
  $$\begin{aligned} \phi (h)=h+O(h^2),\ \text{as}\ h\rightarrow 0. \end{aligned}$$
- The nonlinear function $\theta$ is approximated by nonlocal way.

The major advantages of the aforesaid method are given as (see details^33^):

- The NSFD method avoids numerical instabilities even on using very small step size we need to implement the scheme.
- First, the NSFD method behaves more or equally well with an order RK4 technique most of the time. Although the numerical algorithmic complexity is similar to that of the Euler scheme, there is a significant computational benefit.
- Second, a system that provides good dynamic behavior even for huge time increments is produced by maintaining the dynamical restrictions. Additionally, this offers a significant computational and implementation advantage.

To deduce the NSFD algorithm for our model (2). We can write in differences form $S^{\prime }$ as

27 $$\begin{aligned} S^{\prime }= & {} \frac{S^{k+1}-S^k}{\phi (h)},\ E^{\prime }=\frac{E^{k+1}-E^k}{\phi (h)}, \ I^{\prime }_{S}=\frac{I_{S}^{k+1}-I_{S}^k}{\phi (h)}, \end{aligned}$$

28 $$\begin{aligned} I_{A}^{\prime }= & {} \frac{I_{A}^{k+1}-I_{A}^k}{\phi (h)},\ R^{\prime }=\frac{R^{k+1}-R^k}{\phi (h)}, \ P^{\prime }=\frac{P^{k+1}-P^k}{\phi (h)}. \end{aligned}$$

Using (27) in (2), we get

29 $$\begin{aligned} \frac{S^{k+1}-S^k}{\zeta (h)}&=b-\frac{\beta _{1}S^{k+1}P^k}{1+\alpha _{1}P^k}-\frac{\beta _{2} S^{k+1}(I^{k}_{A}+I^{k}_{S})}{1+\alpha _{2}(I^{k}_{A}+I^{k}_{S})}+\varphi R^{k}-\mu S^{k+1},\\ \frac{E^{k+1}-E^k}{\zeta (h)}&=\frac{\beta _{1}S^kP^k}{1+\alpha _{1}P^k}+\frac{\beta _{2} S^k(I^k_{A}+I^k_{S})}{1+\alpha _{2}(I^k_{A}+I^k_{S})}-(\mu +\upsilon )E^{k+1},\\ \frac{I_{S}^{k+1}-I_{S}^k}{\zeta (h)}&=\eta \upsilon E^k-(\mu +\delta +\gamma _{S}+\tau _{S})I^{k+1}_{S},\\ \frac{I_{A}^{k+1}-I_{A}^k}{\zeta (h)}&=(1-\eta )\upsilon E^k-(\mu +\delta +\gamma _{A}+\tau _{A})I^{k+1}_{A},\\ \frac{R^{k+1}-R^k}{\zeta (h)}&=\gamma _{A}I^k_{A}+\gamma _{S}I^{k}_{S}-(\mu +\varphi ) R^{k+1},\\ \frac{P^{k+1}-P^k}{\zeta (h)}&=\tau _{A}I^k_{A}+\tau _{S}I^k_{S}-\mu _{P}P^k, \end{aligned}$$

such that $\zeta (h)=1-\exp (-h)$ (see^36^), and $h\rightarrow 0$, then $\zeta (h)\rightarrow 0$. After rearranging the terms, we get the algorithm in simplified form as

30 $$\begin{aligned}{}&S^{k+1}=\frac{S^k+\zeta (h)(b+\varphi R^k)}{1+\zeta (h)\left[\frac{\beta _{1}P^k}{1+\alpha _{1}P^k}-\frac{\beta _{2} (I^{k}_{A}+I^{k}_{S})}{1+\alpha _{2}(I^{k}_{A}+I^{k}_{S})}+\mu \right]},\\&E^{k+1}=\frac{1}{1+\zeta (h)(\mu +\upsilon )}\left[\frac{\beta _{1}S^kP^k}{1+\alpha _{1}P^k}+\frac{\beta _{2} S^k(I^k_{A}+I^k_{S})}{1+\alpha _{2}(I^k_{A}+I^k_{S})} \right],\\&I_{S}^{k+1}=\frac{1}{1+\zeta (h)(\mu +\delta +\gamma _S+\tau _S)}\left[I_{S}^k+\zeta (h)(\eta \upsilon E^k) \right],\\&I_{A}^{k+1}=\frac{1}{1+\zeta (h)(\mu +\delta +\gamma _A+\tau _A)}\left[I_{A}^k+\zeta (h)((1-\eta ) \upsilon E^k) \right],\\&R^{k+1}=\frac{1}{1+\zeta (h)(\mu +\varphi )}\left[I_{S}^k+\zeta (h)(\gamma _{A}I^k_{A}+\gamma _{S}I^{k}_{S}) \right],\\&P^{k+1}=\frac{1}{1+\zeta (h)\mu _P}\left[P^k+\zeta (h)(\tau _{A}I^k_{A}+\tau _{S}I^k_{S}) \right]. \end{aligned}$$

From the numerical scheme we get (30), the positivity condition of the NSFD method clearly can be observed. We use the said method and the following numerical values given in Table 3 for the simulations to present graphically our results.

**Table 3:** For the nomenclatures of model (2), the used values.

Nomenclature	Numerical Values	Source	Nomenclature	Numerical Values	Source
*S*(0)	93,000	^1^	$\delta$	0.0018	^1^
*E*(0)	1000	^1^	$\alpha _1$	0.10, 0.05, 0.15	^1^
$I_{S}(0)$	50	^1^	$\beta _{1}$	0.00414	^1^
$I_{A}(0)$	50	^1^	$\beta _{2}$	0.0115	^1^
*R*(0)	0	^1^	$\gamma _{S}$	0.05 per day	^1^
*P*(0)	500	^1^	$\gamma _{A}$	0.0714 per day	^1^
*b*	0.00018	^1^	$\tau _{S}$	0.1 per day	^1^
$\mu$	0.00004563 per day	^1^	$\tau _{A}$	0.05 per day	^1^
$\mu _{P}$	0.1724 per day	^1^	$\varphi$	0.0051	^1^
$\upsilon$	0.09	^1^	$\alpha _2$	0.10, 0.05, 0.15	^1^
$\eta$	0.7	^1^	*b*	0.00018 per day	^1^

**Figure 3:**
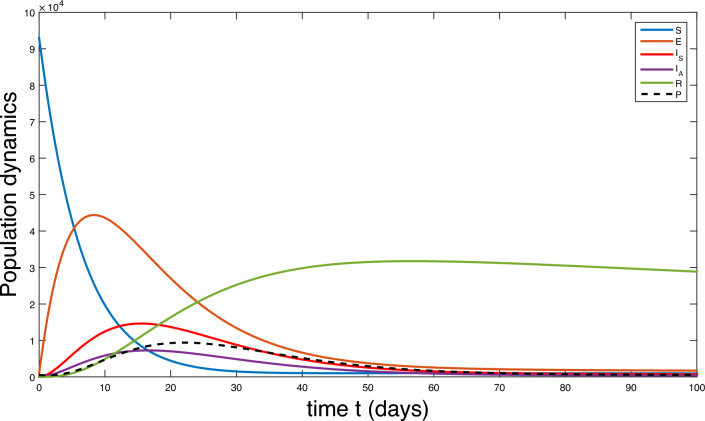
The population dynamics of the proposed model (2).

The population dynamics is presented graphically in Fig. 3 using the numerical values of Table 3. Here we present the population using the values for the parameters $\alpha _{1}=0.1,\ \alpha _2=0.1$.

Here in Fig. 3 we have displayed the combined population dynamics for the given parameters values.

### Case I

**Figure 4:**
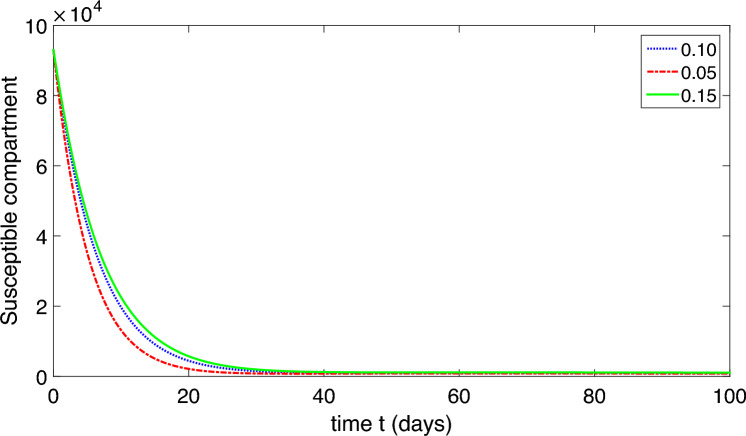
Dynamics of the susceptible class of the proposed model (2).

**Figure 5:**
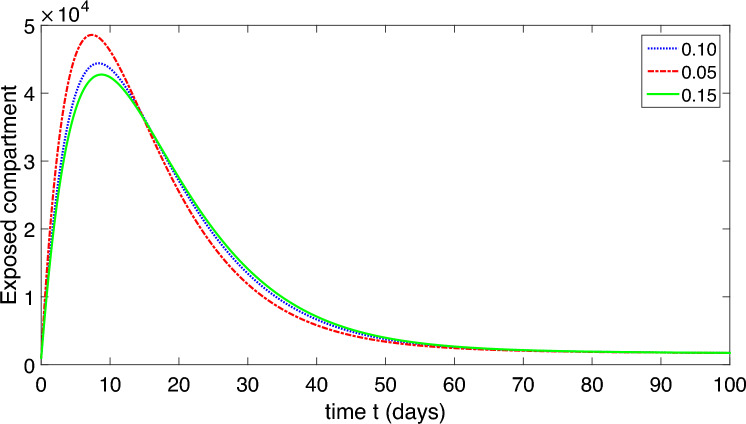
Dynamics of the exposed class of the proposed model (2).

**Figure 6:**
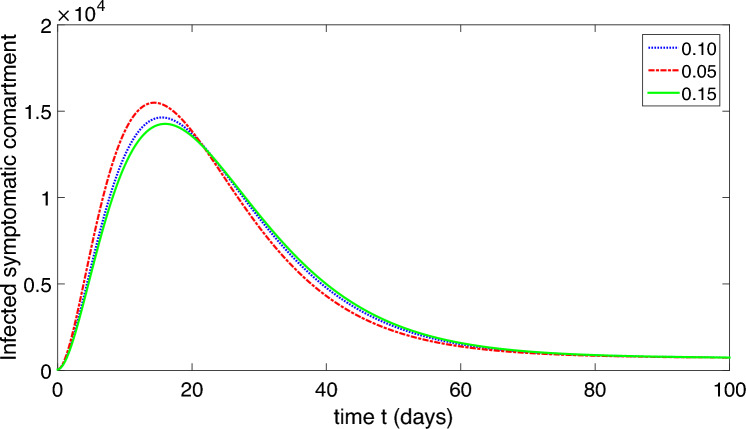
Dynamics of the symptomatic infected compartment of the proposed model (2).

**Figure 7:**
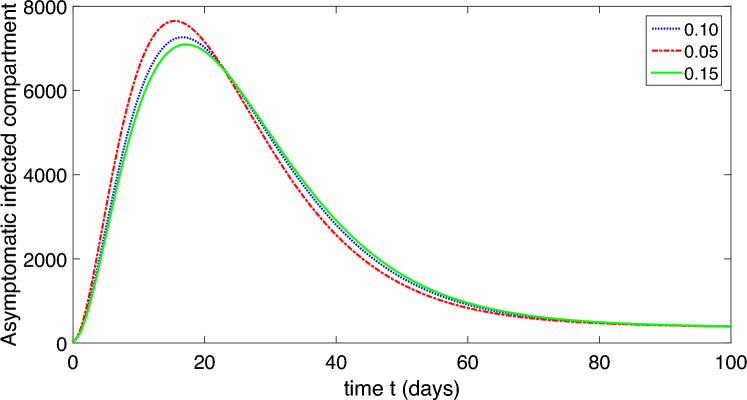
Dynamics of the asymptomatic infected compartment of the proposed model (2).

**Figure 8:**
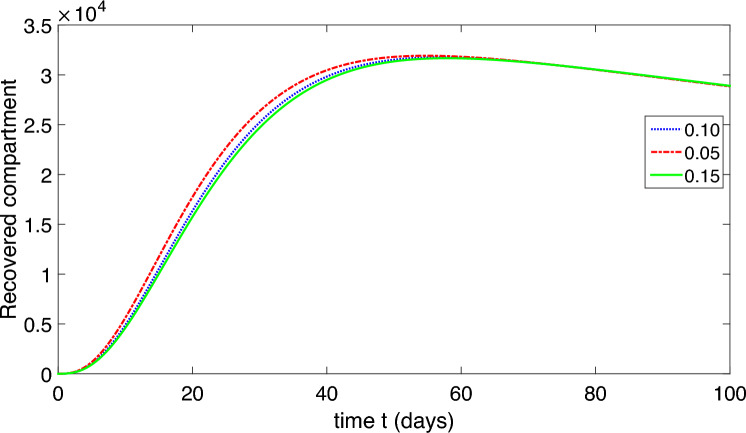
Dynamics of the recovered class of the proposed model (2).

**Figure 9:**
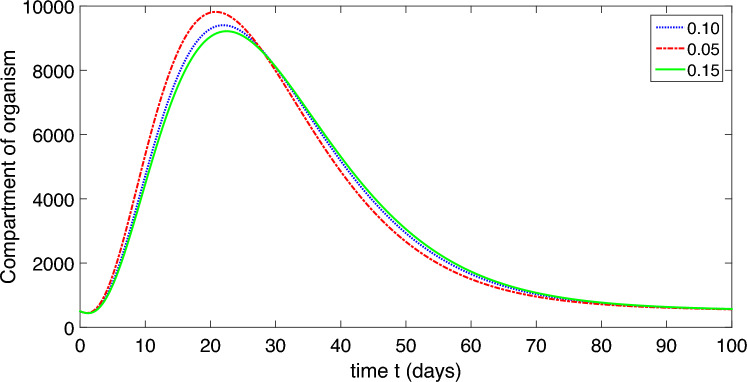
Dynamics of the compartment of organism that causes disease of the proposed model (2).

When $\alpha _1=0.05, \ 0.10, \ 0.15$, we describe the population dynamics of various classes using that values of $\alpha _2=0.10$ in Figs. 4, 5, 6, 7, 8, 9.

Here in Fig. 4 as time increases from 0 to 100 days in the populations. The number of susceptible people decreases quickly over the first ten days while the number of exposed people rises quickly in first 20 days as a result of contact with infected people as shown in Fig. 5. Also the population of infected people is increasing very rapidly for initial 20 days. In the same way there is quick increase of symptomatic infected and asymptomatic infected classes (see Figs. 6, and 7). The concentration of pathogen is also increases as shown in Fig. 9. Also the population of recovered class is rising smoothly as in Fig. 8. Here as the infectious environment ratio increases, therefore for first 25 days, the spread of COVID-19 through interactions in homes, schools, workplaces, restaurants, or during commutes increases. After the 25 days decline can be seen in the transmission dynamics. When $\alpha _1=0.05$ there is high chance to get infection, similarly if we increase $\alpha _1=0.10$ the chance further decreases to get infection and so on. For $\alpha _1=0.15$, there is further low chance of getting infection in contact with infectious environment like institutions, market, food shops etc.

### Case II

**Figure 10:**
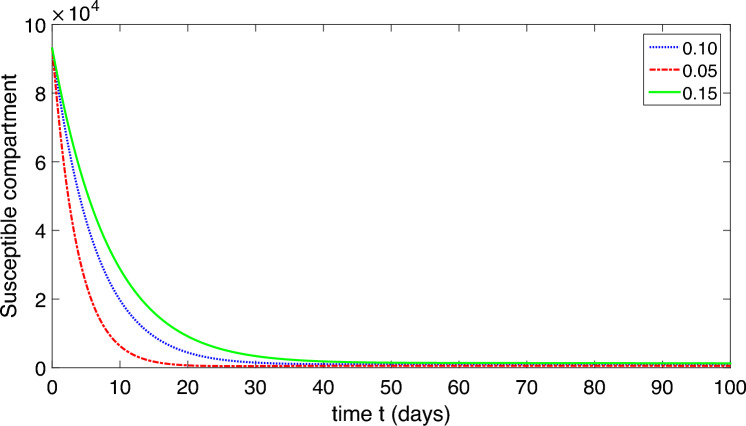
Dynamics of the susceptible class of the proposed model (2).

**Figure 11:**
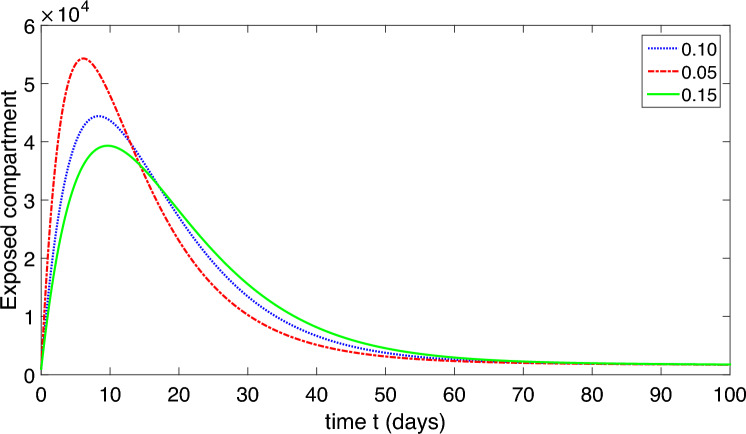
Dynamics of the exposed class of the proposed model (2).

**Figure 12:**
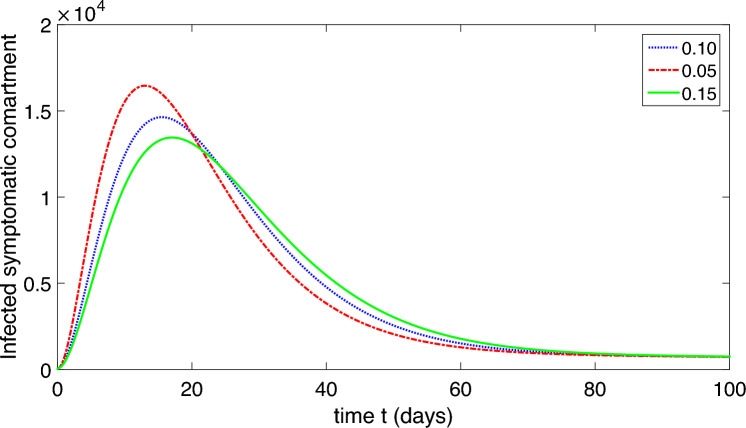
Dynamics of the Infected symptomatic infected compartment of the proposed model (2).

**Figure 13:**
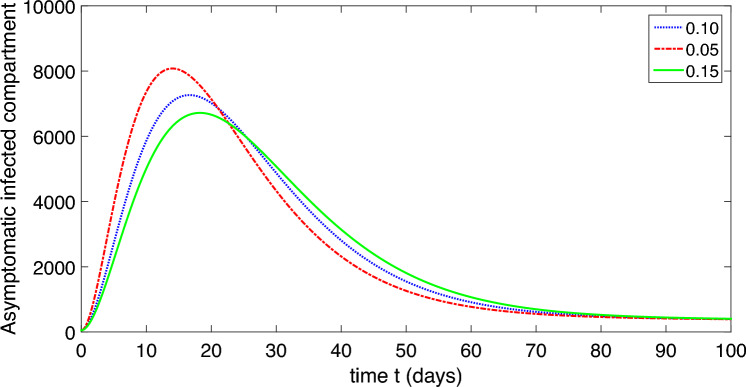
Dynamics of the asymptomatic infected compartment of the proposed model (2).

**Figure 14:**
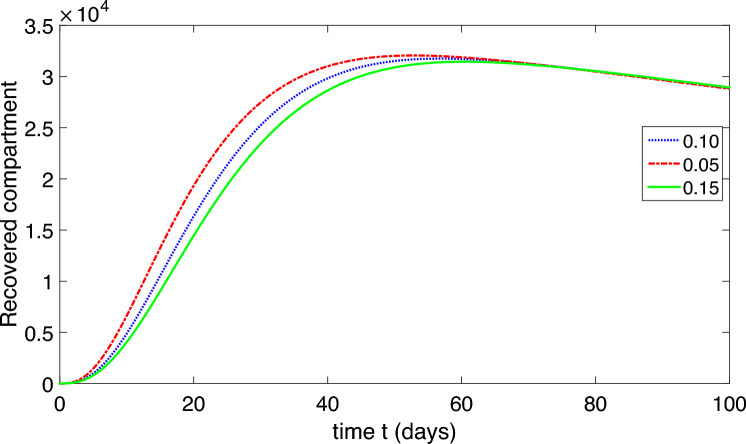
Dynamics of the recovered class of the proposed model (2).

**Figure 15:**
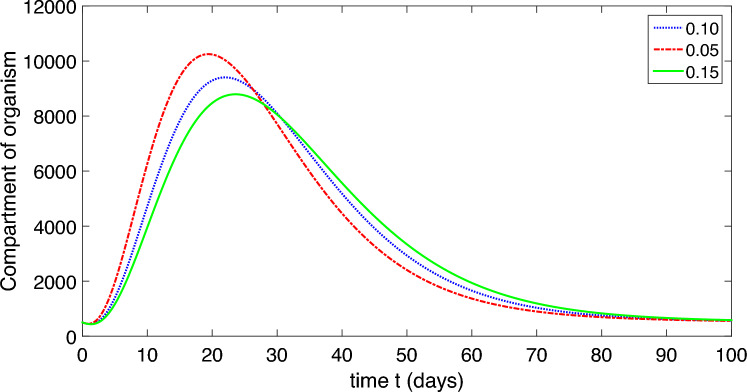
Dynamics of the compartment of organism that causes disease of the proposed model (2).

When $\alpha _2=0.05, \ 0.10, \ 0.15$, we describe the population dynamics of various classes using that values of $\alpha _1=0.10$ in Figs. 10, 11, 12, 13, 14, 15.

As time increases in the populations from 0 to 100 days, Fig. 10 shows this. As illustrated in Fig. 11, the number of susceptible individuals rapidly drops during the first ten days, while the number of exposed individuals rapidly increases during the first twenty days as a result of interaction with infected individuals. For the first 20 days after infection, the number of infected individuals is also growing quite quickly. Similarly, both symptomatic and asymptomatic infected classes are rapidly growing (see Figs. 12 and 13). As seen in Fig. 15, there is also a rise in pathogen concentration. As seen in Fig. 14, the population of the recovered class is likewise growing steadily. Here, for the first 25 days, the spread of diseases increases as the infectious environment ratio does. On reducing the social interaction with infectious individual, the chances of getting infection is also rising. As at $\alpha _2=0.05$, there is high risk to get infection. But on increasing the values of $\alpha _2=0.10$, and $\alpha _2=0.15$, the social distance increases the chances of getting infection will decrease.

## Conclusion

The present article conducts a comprehensive review aimed at understanding the global dynamics of COVID-19. The researchers identified equilibrium points, calculated the basic reproductive rate, and assessed the model’s stability at disease-free and endemic equilibrium states, both locally and globally. The main focus lies in examining COVID-19 dynamics using a suggested model, with particular attention to the characteristics of V–L matrices. A series of lemmas and theorems based on V–L matrices theory are presented in this regard to study the global stability of the endemic equilibrium. Non-standard finite difference techniques are employed for numerical analysis, contributing to ongoing efforts to comprehend the pandemic’s intricacies. The paper also elaborates on the effects of social distance and interaction with infected environments. Precautionary measures such as increasing social distances, wearing face masks, sanitation, and avoiding large gatherings are highlighted as effective strategies for reducing the risk of infection. These findings contribute to the broader understanding of COVID-19 dynamics and provide insights into potential strategies for mitigating its spread.

As future work, the authors plan to utilize the Volterra–Lyapunov (V–L) matrix theory approach to investigate the global stability of other infectious disease models that have not yet been thoroughly studied using the said methodology.

## Data Availability

All the data used is included in the paper.
